# CFTR Modulation by *Plantago asiatica* Flavonoids: A Potential Antisecretory Mechanism Against Diarrhoea

**DOI:** 10.3390/membranes16100308

**Published:** 2026-09-22

**Authors:** Wenqiang Cui, JiaHao Liu, Ruiguang Wang, Yongzhi Ren, Yanhua Li

**Affiliations:** 1 College of Veterinary Medicine, Northeast Agricultural University, Harbin 150030, China; 2 Heilongjiang Key Laboratory for Animal Disease Control and Pharmaceutical Development, Harbin 150030, China

**Keywords:** *Plantago asiatica*, CFTR, secretory diarrhoea, antidiarrhoeal activity, molecular dynamics

## Abstract

*Plantago asiatica* L. (*P. asiatica*) is traditionally used to treat diarrhoea in veterinary medicine, but its molecular basis remains unclear. In rodent models, the water extract and active fractions reduced intestinal fluid accumulation without significant transit inhibition by the crude extract, supporting a substantial antisecretory component. A phenotype-guided analysis of 14 compounds identified from the active fractions prioritised cystic fibrosis transmembrane conductance regulator (CFTR), the principal apical chloride channel driving intestinal fluid secretion. Apigenin, luteolin and scutellarein were then evaluated at the lipid-exposed ivacaftor site of porcine CFTR in a POPC membrane. Although this regulatory site can support both CFTR potentiation and inhibition, the ligand-specific binding arrangements underlying these opposite effects remain unclear. Luteolin formed a persistent Glu874-centred network and produced the largest reproducible shift in local TM8 hinge geometry, providing a structural hypothesis for inhibitory modulation. Apigenin sampled a TM4-shifted State A and a TM8-facing State B that overlapped the ivacaftor-associated pocket. In light of its reported concentration-dependent block and potentiation, these ensembles are proposed as inhibition-associated and potentiation-associated modes, respectively. Scutellarein remained associated with the shared pocket and exhibited a distinct interaction pattern, further supporting the ligand-dependent conformational plasticity of this regulatory site. These findings support CFTR modulation by *P. asiatica* flavonoids as a plausible mechanism for reducing chloride-driven intestinal fluid secretion.

## 1. Introduction

Diarrhoea remains an important health problem in both humans and livestock. In veterinary practice, its management commonly combines fluid and electrolyte replacement, dietary and hygiene measures and, where indicated, cause-directed antimicrobial or antiparasitic treatment. Concerns regarding antimicrobial resistance and the limitations of nonspecific antimotility treatment have encouraged the development of non-antibiotic approaches targeting intestinal fluid secretion [1,2]. Antidiarrhoeal treatments may act by reducing intestinal motility, limiting intestinal fluid secretion, or combining both effects. These mechanisms should be distinguished because slowing intestinal transit does not necessarily correct the excessive epithelial secretion that drives secretory diarrhoea. Chloride transport across the apical membrane of intestinal epithelial cells is a major determinant of luminal fluid accumulation [2,3,4,5]. The cystic fibrosis transmembrane conductance regulator (CFTR) is an epithelial chloride channel that plays a central role in this process. Excessive intestinal CFTR activity promotes chloride and water secretion, whereas pharmacological CFTR inhibition reduces toxin-induced fluid accumulation [2,6,7,8]. CFTR is therefore an important membrane target for antisecretory intervention. CFTR activity is regulated by phosphorylation, ATP binding, and conformational changes within its transmembrane domains [9,10]. Structural studies have identified a lipid-exposed regulatory site formed mainly by transmembrane helices TM4, TM5, and TM8, where the clinically used potentiator ivacaftor binds and promotes channel opening [11,12,13,14]. More recent structure-guided work identified both potentiators and inhibitors acting through the same ivacaftor-defined site [15]. However, structural characterisation was achieved for a potentiating complex, whereas an inhibitor-bound structure at this site was not resolved. It therefore remains unclear how different ligand-binding arrangements within this shared pocket produce opposite effects on CFTR gating [10,15].

*Plantago asiatica* L. (*P. asiatica*) provides two distinct medicinal materials: the dried whole plant (Plantaginis Herba; Cheqian-cao) and the seeds (Plantaginis Semen; Cheqian-zi) [16]. The seeds contain water-swelling mucilage that can increase stool viscosity and modify stool consistency through a physicochemical mechanism [17]. In contrast, the present study investigated a hot-water extract of the dried whole plant, consistent with the documented preparation of whole-plant *P. asiatica* as an oral decoction [18]. The aqueous preparation was selected to approximate a conventional herbal decoction rather than a seed-mucilage preparation. The dried whole plant contains diverse secondary metabolites, including flavonoids, phenylethanoid glycosides, triterpenoids and phenolic acids [19,20]. In this context, *P. asiatica* represents a traditionally used and readily prepared source of constituents with potential antisecretory activity, providing a complementary non-antibiotic strategy distinct from conventional antimicrobial and antimotility treatments. Previous chemical profiling identified 14 compounds in its active fractions, but the relationship between these constituents, the observed antidiarrhoeal phenotype and a membrane-transport target remains insufficiently defined. Ligand-based target prediction and disease-association databases provide a means of prioritising candidate targets from chemically characterised plant extracts. SwissTargetPrediction estimates potential protein targets from chemical structure, whereas GeneCards integrates available information on genes associated with human diseases [21,22]. Such predictions require interpretation in the context of the observed pharmacological phenotype and existing experimental evidence. In this respect, previous functional studies showed that apigenin produces concentration-dependent potentiation and a block of the CFTR, whereas luteolin inhibits CFTR-mediated chloride secretion [23,24]. Nevertheless, the binding modes underlying these different effects remain unknown.

In this study, we first evaluated the effects of the *P. asiatica* water extract and its partition fractions on experimental diarrhoea, intestinal transit, and intestinal fluid accumulation. These complementary in vivo assays were required to distinguish effects on intestinal fluid secretion from general inhibition of gastrointestinal motility, which cannot be assessed together in a single in vitro or ex vivo model. The pharmacological phenotype was then used to guide target prioritisation for the 14 previously reported compounds. Compounds associated with the selected membrane-transport target were subsequently examined by molecular docking and membrane-embedded molecular dynamics simulations. A porcine CFTR model was used to maintain relevance to the veterinary context, and structure-derived ivacaftor served as the reference ligand. This integrated approach was designed to connect the antidiarrhoeal phenotype of *P. asiatica* with ligand-specific recognition of a membrane target controlling intestinal chloride and fluid secretion.

## 2. Materials and Methods

### 2.1. Plant Material, Animals and Ethical Approval

Male and female Kunming mice (18–22 g) and Wistar rats (180–200 g) were obtained from the Animal Center of Harbin Medical University (Harbin, China). Animals were housed in plastic cages at 22 ± 2 °C with free access to standard pellet diet and water and were acclimatised for at least 3 days. Dried whole-plant material of *P. asiatica* (Plantaginis Herba; reference no. 3777555) was purchased from the Harbin Traditional Chinese Medicine Market, Heilongjiang, China. Plantaginis Herba is medicinally distinct from Plantaginis Semen, the seed-derived material [16]. Chemicals were of analytical grade and were obtained from Solarbio (Beijing, China) unless otherwise specified. Animal procedures were approved by the Ethics Committee for Experimental Animal Research of Northeast Agricultural University (approval no. NEAUEC20). Animals were euthanised by sodium phenobarbital injection after the experiments.

### 2.2. Preparation and Fractionation of the Water Extract

Water extraction was selected to approximate the general preparation principle of a traditional herbal decoction and to generate an orally administrable crude preparation for in vivo evaluation. Powdered *P. asiatica* (100 g) was mixed with 1 L distilled water, boiled for 2 h and filtered through gauze. The distilled water used in this study was not subjected to additional purification. Extraction was repeated three times. The combined filtrates were concentrated to approximately 100 mL using an RE-52A rotary evaporator (Shanghai Yarong Biochemical Instrument Factory, Shanghai, China). The concentrated extract was suspended in distilled water and successively partitioned with petroleum ether, chloroform, ethyl acetate and *n*-butanol. Solvent fractions were concentrated under reduced pressure and dried in a thermostatic forced-air oven. Petroleum-ether (PEF), chloroform (CF), ethyl-acetate (EAF), *n*-butanol (BuF) and remaining aqueous fractions (RFs) were stored at 4 °C before use.

### 2.3. In Vivo Antidiarrhoeal Assays

For castor-oil-induced diarrhoea, Kunming mice were randomly assigned to five groups (n = 6). The negative control received distilled water (0.2 mL, oral), the positive control received loperamide hydrochloride (2 mg kg^−1^, oral), and the test groups received water extract at 150, 300 or 450 mg kg^−1^. One hour later, each mouse received 0.2 mL castor oil orally and was housed individually over pre-weighed absorbent paper for 6 h. Diarrhoea-onset time and wet-faeces number were recorded.

For the charcoal-meal assay, mice were fasted for 12 h with access to water. One hour after distilled water, atropine sulphate (2 mg kg^−1^) or test-sample administration, mice received charcoal meal orally. Thirty minutes later, the small intestine was removed from pylorus to caecum, and transit was calculated as charcoal migration distance divided by total intestinal length. The percentage was calculated separately for each animal, and group results are reported as mean ± SD (n = 6).

For intestinal-fluid accumulation, Wistar rats were fasted for 12 h with access to water. After distilled water, atropine sulphate (2 mg kg^−1^) or test-sample administration, each rat received 1 mL castor oil orally. After 30 min, intestinal content volume and weight were measured. Fraction activity was evaluated with the same assays and the doses reported in Tables S4–S6. Fraction doses are expressed as milligrams of the dried isolated fraction per kilogram of body weight and are not crude-extract-equivalent doses.

### 2.4. Compound Identification and Target Prioritisation

Fourteen compounds were identified by UPLC-Q-TOF/MS as reported previously [19]. Canonical structures were submitted to SwissTargetPrediction [21] for *Homo sapiens* (accessed 7 August 2026). The initial SwissTargetPrediction output comprised 1400 ranked compound–target records, corresponding to 100 predictions for each of the 14 compounds. Of these, 357 records met the probability threshold of ≥0.9. After duplicate target entries across compounds were merged, 137 unique target entities remained. Five multi-protein complex entries lacking a unique gene symbol were excluded before GeneCards matching, yielding 132 gene-mappable high-probability targets. These targets were then intersected with genes returned by a GeneCards [22] search for “diarrhea” (accessed 7 August 2026), using a relevance score ≥ 20, consistent with published network-pharmacology precedent [25]. SwissTargetPrediction probability and GeneCards relevance were applied sequentially and were neither normalised nor combined into a composite score. The 37 intersecting targets were ranked directly by their GeneCards relevance scores, and the compound–target network was visualised in Python 3.10.14 using NetworkX 3.4.2 and Matplotlib 3.9.2. CFTR was prioritised for structural analysis because its high disease-association rank was accompanied by a direct mechanistic link to intestinal fluid secretion; the ranking was used as a prioritisation tool rather than as evidence of target exclusivity. Among the 14 compounds, apigenin, luteolin and scutellarein were the three compounds predicted to target CFTR at the retained probability threshold and were therefore selected for structural analysis.

### 2.5. Porcine CFTR Model and Molecular Docking

A porcine CFTR homology model was prepared in Schrödinger Maestro from the porcine UniProt sequence Q6PQZ2 and aligned to phosphorylated, ATP-bound, ivacaftor-bound human CFTR (PDB 6O2P) [12]. Porcine CFTR was selected to reflect the veterinary and livestock context of this study. Porcine CFTR shares approximately 92% full-length amino-acid sequence identity with human CFTR, supporting the use of the human 6O2P structure as a template [26]. Missing loops were not rebuilt; chain terminations were retained after residues 409, 637 and 1174. Two ATP molecules and their associated Mg^2+^ ions were retained. The structural quality of the prepared porcine CFTR model was evaluated using the SAVES structure-validation suite (https://saves.mbi.ucla.edu/). Backbone stereochemistry was assessed using a PROCHECK Ramachandran plot [27] and the consistency of non-bonded atomic interactions was evaluated using ERRAT [28].

The protein was processed using the Protein Preparation Wizard in Schrödinger Maestro (release 2025-4; Schrödinger, LLC, New York, NY, USA). Hydrogen atoms were assigned for physiological pH and restrained minimisation used OPLS4 with a 0.3 Å heavy-atom RMSD limit. Apigenin, luteolin and scutellarein were prepared with LigPrep and docked with Glide in standard-precision mode. The structure-derived ivacaftor molecule was removed before flavonoid docking and its binding position was used only to define the docking grid [29]. Ligand flexibility was retained, and poses without major steric clashes that occupied the ivacaftor-defined region were selected for membrane simulation. Ivacaftor was not re-docked.

### 2.6. Membrane-System Construction and Parameterisation

The complexes were oriented according to the OPM orientation of 6O2P [30] and embedded with PACKMOL-Memgen [31] in a POPC bilayer containing 124 lipids per leaflet. The system was completed with PACKMOL [32], OPC water and approximately 0.15 M KCl. Each final system contained porcine CFTR, two ATP, two Mg^2+^ ions, one small-molecule ligand, 248 POPC molecules, 71,146 waters, 193 K^+^ ions and 217 Cl^−^ ions. The systems were initially constructed in an orthorhombic periodic cell of 124 × 124 × 204 Å. Following membrane equilibration, the lateral cell dimensions ranged from 105.08 to 105.79 Å, and the dimensions along the membrane normally ranged from 234.63 to 238.13 Å. The lateral dimensions of the CFTR model were approximately 78.11 × 70.04 Å, leaving minimum protein-image separations of approximately 27.0 and 35.0 Å along the two membrane-plane directions. These distances exceeded the 10 Å non-bonded cutoff used in the simulations and prevented direct contact between CFTR and its periodic images. System-specific atom counts and equilibrated cell dimensions are provided in Table S17, and representative system views are shown in Figure S3.

Protein, lipid and ligand parameters were ff19SB [33], Lipid21 [34] and GAFF2/AM1-BCC [35,36], respectively; OPC water [37] and the ATP polyphosphate parameters of Meagher et al. [38] were used. Systems were assembled with tleap from AmberTools 24, simulated with AMBER 25 [39], and processed with cpptraj from AmberTools 25.

### 2.7. Molecular Dynamics Protocol

Each system underwent three minimisation stages, 1 ns NVT heating from 0 to 300 K, and six membrane-equilibration stages totalling 21 ns. Membrane equilibration and production used semi-isotropic NPT pressure scaling (ntp = 3) and constant-surface-tension settings (csurften = 3, ninterface = 2). Three production trajectories were executed per ligand. All production runs began from the same equilibrated eq6.rst7 restart with irest = 1 and ntx = 5; they therefore inherited the same starting velocities. Accordingly, these simulations are described as separately propagated trajectories rather than statistically independent replicas. Production was performed with pmemd.cuda. The complete 0–120 ns interval of each trajectory was used, giving 360 ns per ligand and 1.44 μs in total.

### 2.8. Trajectory Processing and State-Resolved Analysis

Periodic-boundary processing and trajectory sampling were performed with cpptraj [40]. One thousand uniformly spaced frames (120 ps spacing) were analysed for each trajectory. CFTR RMSD was calculated from CA/C/N/O backbone atoms after fitting to the first frame. Ligand RMSD used ligand heavy atoms after protein fitting. Ligand–protein contacts were defined by a 4.5 Å heavy-atom cutoff. Hydrogen bonds used 3.0 Å donor–acceptor distance and 135° angle cutoffs.

State-resolved calculations used MDAnalysis 2.4.3 [41,42] and SciPy. Protein-aligned apigenin heavy-atom coordinates were partitioned by deterministic two-state k-means clustering. The cluster closest to the first production frame was designated State A, and the other major cluster State B. State assignment was therefore based on clustered ligand coordinates rather than ligand RMSD alone. The resulting ensembles were further characterised using residue-specific contact occupancies, hydrogen-bond occupancies, representative ligand orientations and TM8 hinge geometry. State-specific contact occupancies were calculated using only frames assigned to the corresponding state. A sustained transition to State B was defined as the first of 20 consecutive State B frames, corresponding to 2.4 ns. Real trajectory frames nearest the cluster centroids were selected as representative structures. Ligand–protein contacts were defined using a 4.5 Å heavy-atom cutoff. The main-text heat map retained the 16 residues with the highest score, defined as maximum occupancy plus 1.5 times the between-ensemble variance; complete values are reported in Table S15. The TM8 hinge angle was calculated from Cα segment vectors flanking native porcine residues 926–934 and summarised at the trajectory level. Native porcine CFTR residue numbering is used throughout, and Table S14 provides the mapping to the consecutively numbered simulation topology.

### 2.9. Statistical Analysis

Animal results are reported as mean ± SD. Statistical analyses were performed using SPSS 13.0. Group comparisons were performed using analysis of variance followed by LSD multiple-comparison tests; *p* < 0.05 was considered statistically significant.

## 3. Results

### 3.1. The Water Extract Reduces Diarrhoea and Intestinal Fluid Accumulation Without Significantly Altering Transit

The water extract of *P. asiatica* significantly reduced diarrhoeal output, with progressively greater numerical effects across the tested doses. The number of wet faeces decreased from 10.7 ± 1.02 in the negative-control group to 7.3 ± 0.98, 6.5 ± 0.56 and 5.6 ± 0.15 per 6 h at 150, 300 and 450 mg/kg, respectively, corresponding to reductions of 31.78%, 39.25% and 47.66% (Figure 1A; Table S1). By contrast, intestinal transit was not significantly altered by the water extract, with transit rates of 69.31 ± 1.87%, 66.26 ± 2.57% and 62.42 ± 1.72%, compared with 71.98 ± 2.68% in the negative-control group (Figure 1B; Table S2). Intestinal fluid volume was significantly reduced from 3.24 ± 0.02 mL in the negative-control group to 2.01 ± 0.06, 1.89 ± 0.05 and 1.52 ± 0.02 mL, corresponding to inhibition rates of 37.96%, 41.67% and 53.09%, respectively (Figure 1C; Table S3). The positive controls produced the expected responses: loperamide prolonged diarrhoea onset and reduced wet faeces by 51.40%, whereas atropine inhibited intestinal transit and fluid accumulation by 51.71% and 57.10%, respectively (Tables S1–S3). Thus, the water extract reduced diarrhoea and intestinal fluid accumulation without producing a statistically significant inhibition of intestinal transit, supporting a predominantly antisecretory effect.

### 3.2. Antidiarrhoeal Activity Is Concentrated in the Ethyl Acetate and Remaining Fractions

Because the fractionation experiments were conducted with separate concurrent negative controls, their effects were expressed as percentages relative to the corresponding control group. The ethyl acetate fraction (EAF) produced progressively greater protection against castor-oil-induced diarrhoea across the tested doses of 43.86%, 54.39% and 69.30% at 150, 300 and 450 mg/kg, respectively, whereas the corresponding values for the remaining fraction (RF) were 34.78%, 46.12% and 55.58% (Figure 1D; Table S4). The EAF and RF also showed progressively greater numerical effects on intestinal transit and intestinal fluid accumulation across the tested doses (Figure 1E,F; Tables S5 and S6). In contrast, the petroleum ether, chloroform and *n*-butanol fractions showed little or no significant activity. These results indicate that the major antidiarrhoeal constituents were concentrated in the EAF and RF. However, unlike the crude water extract, both active fractions affected intestinal transit as well as fluid accumulation, suggesting that fractionation separated constituents with partially overlapping antisecretory and motility-related effects.

### 3.3. Target Prediction Combined with Disease-Association Ranking Prioritises CFTR as a Candidate Target

The 14-compound active-fraction dataset comprised flavonoids, flavonoid glycosides, phenylethanoid glycosides, a triterpenoid and phenolic acids (Table 1). These compounds were treated as a chemically defined candidate set for target prioritisation rather than as constituents proven individually to account for the activity of the complete water extract. SwissTargetPrediction for *Homo sapiens* retained 132 targets at probability ≥ 0.9. Intersection with GeneCards diarrhoea-associated genes at a relevance score ≥ 20 yielded 37 targets (Table S7A,B). Ten of the 14 compounds contributed at least one target to the final 37-target set. CFTR ranked first (117.84, the GeneCards relevance score for the CFTR–diarrhoea association), ahead of EGFR (114.60), and directly matched the fluid-accumulation phenotype (Figure 2). No statistical or biological significance was assigned to this numerical difference; CFTR was prioritised because of its direct mechanistic relationship with intestinal chloride and fluid secretion. Apigenin, luteolin and scutellarein were the three compounds associated with CFTR at the retained probability threshold and were therefore advanced to structural analysis. The human-centred target databases and subsequent use of a porcine CFTR model are addressed as a cross-species limitation.

### 3.4. Molecular Docking at the Ivacaftor Site

Structural validation supported the quality of the porcine CFTR model. Ramachandran analysis placed 92.6% of the evaluated residues in the most favoured regions and 7.4% in additionally allowed regions, with no residues in disallowed regions (Figure S1). The ERRAT Overall Quality Factor was 85.30, supporting satisfactory non-bonded atomic interactions within the model (Figure S2). Apigenin, luteolin and scutellarein were docked into a porcine CFTR homology model constructed from the ivacaftor-bound structure 6O2P [12]. The structure-derived ivacaftor position was retained without re-docking and defined the comparison site. The domain organisation of CFTR and the structural regions retained in the simulation model are shown in Figure 3A. All three flavonoids could be accommodated within the lipid-exposed TM4, TM5, and TM8 region defined by structure-derived ivacaftor (Figure 3B). Ivacaftor was retained in its experimental position without re-docking, whereas docking provided the starting poses for apigenin, luteolin and scutellarein. Docking defined membrane-compatible starting poses for subsequent state-resolved simulations.

### 3.5. Membrane-Embedded Molecular Dynamics

Each porcine CFTR complex was embedded in a 248-POPC bilayer and analysed over three 0–120 ns production trajectories, giving 360 ns per ligand and 1.44 μs in total. One thousand uniformly spaced frames were used per trajectory without an additional production burn-in. Three separately executed trajectories were analysed for each ligand system to evaluate the reproducibility of the observed conformational behaviour (Table S11).

CFTR backbone RMSD was comparable among systems: 1.73 ± 0.31 Å for ivacaftor, 2.02 ± 0.19 Å for luteolin, 1.76 ± 0.06 Å for apigenin and 1.85 ± 0.15 Å for scutellarein (mean ± SD across three trajectories; Figure 4A). Thus, the ligand-dependent differences were not associated with gross CFTR destabilisation.

Ligand RMSD was calculated relative to the first production frame after protein fitting (Figure 4B). Ivacaftor and luteolin remained compact during production (2.07 ± 0.25 and 1.79 ± 0.25 Å), whereas scutellarein repositioned within the membrane-facing pocket (7.36 ± 0.29 Å). Across the three separately propagated trajectories, apigenin transitioned from State A to State B at 52.08, 54.00 and 43.68 ns, respectively. Following these sustained transitions, State B persisted to the final analysed frame for 67.80, 65.88 and 76.20 ns, respectively, without a subsequent return to State A (Figure S4; Table S13B).

State-resolved contact fingerprints distinguished the two apigenin ensembles (Figure 4C). At a contact-occupancy cutoff of 10%, State B shared nine of the 12 ivacaftor-contacting residues (75.0%), whereas State A shared five of 12 (41.7%), demonstrating greater overlap of State B with the ivacaftor interaction environment. The TM4-shifted State A was dominated by Val232, Leu233, Phe236 and Phe305, with additional contact to Leu932. State B lost this TM4 anchor and shifted towards Phe312, Glu874 and the TM8 Gly926–Arg934 region, overlapping the common ivacaftor-associated pocket. Ivacaftor retained its Tyr304/Phe305/Ser308/Phe312 and TM8 contact network, whereas luteolin used a Glu874-centred TM8 network. Representative structures illustrate State A, State B, luteolin and scutellarein in Figure 4E–H. For clarity, Figure 4C shows the 16 residues with the highest combined occupancy and between-ensemble variance; the complete fingerprint is provided in Table S15.

Hydrogen-bond analysis reinforced the stability of luteolin (Table S8). Its Glu874 hydrogen bond persisted in 97.7–98.7% of frames across trajectories, and the Phe312 backbone interaction showed 85.5–89.4% occupancy. Apigenin and scutellarein formed less persistent hydrogen-bond networks.

The TM8 hinge angle showed the clearest ligand-dependent response for luteolin (39.10 ± 1.50° versus 42.64 ± 1.01° for ivacaftor; Figure 4D). Together with its persistent Glu874-centred anchoring, this reproducible TM8 displacement defines a stable inhibition-associated structural ensemble for luteolin, consistent with its experimentally established blockade of CFTR-mediated chloride secretion [24]. Apigenin State A and State B averaged 41.45 ± 0.73° and 42.09 ± 1.71°, respectively, and their TM8 hinge-angle distributions overlapped. State B adopted a TM8-facing position and contact network that more closely overlapped the ivacaftor interaction environment, whereas State A retained a distinct TM4-shifted arrangement. In conjunction with the experimentally established concentration-dependent potentiation and block of CFTR by apigenin [23], these structural ensembles support the hypotheses that State B and State A represent potentiation-associated and block-associated binding modes, respectively. Scutellarein showed a mean angle close to that of ivacaftor (42.09 ± 0.35°) while retaining a distinct contact pattern, indicating recognition of the shared regulatory pocket through a ligand-specific ensemble.

## 4. Discussion

### 4.1. The In Vivo Phenotype Supports an Antisecretory Mechanism

The water extract significantly reduced intestinal fluid accumulation without significantly altering transit, demonstrating an antisecretory component that was not dependent on measurable motility suppression. The EAF and RF retained this fluid-reducing activity but also inhibited intestinal transit, indicating that the active fractions may combine antisecretory and motility-related effects. CFTR was prioritised because it ranked first among the disease-filtered targets and directly links apical chloride transport to intestinal fluid secretion [2,3,4,5,6,7]. Within the previously characterised active-fraction compounds, apigenin, luteolin and scutellarein were selected for structural comparison based on target prediction and available functional evidence, including the reported antidiarrhoeal activity of luteolin and scutellarein and CFTR modulation by apigenin and luteolin [19,23,24].

### 4.2. The Flavonoids Show Distinct Modes of Recognition at One Regulatory Hotspot

The simulations revealed three ligand-specific recognition modes at the same regulatory hotspot. Luteolin formed the most persistent natural-ligand ensemble, centred on Glu874 and TM8, and produced the largest reproducible local hinge displacement. Luteolin has been experimentally demonstrated to dose-dependently block CFTR-mediated chloride secretion in T84 epithelia [24]. The present simulations extend this functional observation by identifying a persistent Glu874-centred interaction network and a distinct TM8 geometry that provide a structural model for luteolin-mediated inhibition.

Apigenin sampled two reproducible environments: a TM4-shifted State A and a TM8-facing State B that converged on the ivacaftor-associated pocket. Scutellarein also occupied this common region, where its larger repositioning and less persistent hydrogen-bond network defined a comparatively dynamic, ligand-specific recognition mode.

### 4.3. An Inhibitory CFTR Framework and State-Dependent Apigenin Modulation

CFTR activation drives chloride-dependent intestinal fluid secretion, whereas pharmacological CFTR inhibition reduces toxin-induced fluid accumulation [2,3,4,5,6,7,8]. The marked reduction in intestinal fluid accumulation, the phenotype-guided prioritisation of CFTR and the experimentally established CFTR-blocking activity of luteolin [24] collectively support inhibitory CFTR modulation as a candidate mechanism contributing to the antisecretory action of *P. asiatica*. The additional effects of the EAF and RF on intestinal transit indicate that the active fractions may combine antisecretory and motility-related actions.

Apigenin provides a complementary, state-dependent mechanism. Direct electrophysiology showed potentiation at lower concentrations and a block at higher concentrations [23]. State B overlaps the ivacaftor-associated TM8-facing ensemble and is therefore compatible with potentiation, whereas the TM4-shifted State A is a plausible block-associated mode. This interpretation is consistent with structural evidence that the same ivacaftor hotspot can accommodate positive and negative allosteric modulators [15].

### 4.4. Limitations and Testable Predictions

The study combines rodent efficacy models, human-centred target databases and a porcine homology model. Atomistic MD provides mechanistic structural ensembles rather than a direct functional readout [43], and trajectory-to-trajectory variability must be considered when interpreting sampled state fractions [44]. Here, the three trajectories per ligand share an equilibrated starting restart, the POPC bilayer is simpler than an enterocyte apical membrane, and 120 ns may not capture slower transitions. Because all apigenin trajectories originated from the same docked pose, an influence of the starting configuration on the early State A ensemble and the observed transition pathway cannot be completely excluded. The proposed functional assignments of the apigenin ensembles should therefore be regarded as testable structural hypotheses rather than direct evidence of channel potentiation or inhibition. The principal predictions are therefore testable by electrophysiological concentration-response measurements, mutagenesis of Glu874 and the state-discriminating TM4/TM8 residues, and competition with ivacaftor.

## 5. Conclusions

*P. asiatica* produced a predominantly antisecretory antidiarrhoeal phenotype, and CFTR was prioritised as the membrane target most directly linked to that response (Figure 5). Luteolin combined persistent Glu874-centred binding with the largest reproducible shift in local TM8 hinge geometry, making it the strongest candidate for inhibitory CFTR modulation. Apigenin sampled a TM4-shifted State A and an ivacaftor-overlapping TM8-facing State B, providing a structural hypothesis for its reported concentration-dependent block and potentiation, respectively. Scutellarein remained associated with the shared site and adopted a distinct recognition pattern, further illustrating the conformational adaptability of this lipid-exposed regulatory pocket. Together, the results support modulation of CFTR-dependent chloride secretion as a plausible mechanism for the antidiarrhoeal action of *P. asiatica* and define specific binding modes for experimental testing.

## Figures and Tables

**Figure 1:**
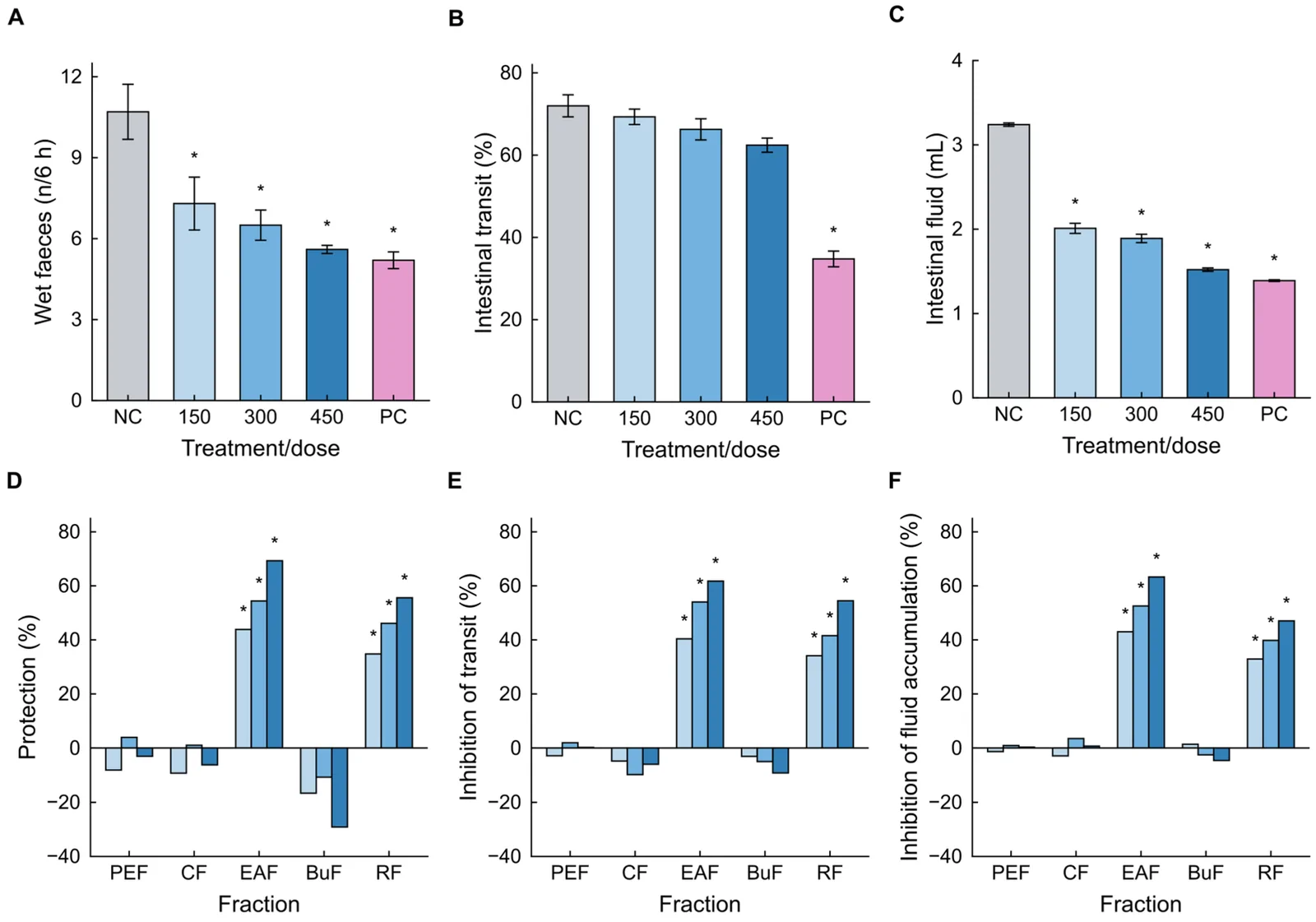
Antidiarrhoeal effects of the water extract and partition fractions of *P. asiatica.* (**A**) Wet faeces during 6 h after castor-oil administration. (**B**) Charcoal-meal intestinal transit. (**C**) Intestinal fluid volume. (**D**–**F**) Protection against diarrhoea, inhibition of transit and inhibition of fluid accumulation, respectively. In (**D**–**F**), light, intermediate and dark blue bars denote 150, 300 and 450 mg kg^−1^. Values in (**A**–**C**) are mean ± SD; percentages in (**D**–**F**) are relative to the concurrent negative control. NC, negative control; PC, positive control; PEF, petroleum ether fraction; CF, chloroform fraction; EAF, ethyl acetate fraction; BuF, n-butanol fraction; RF, remaining fraction. * *p* < 0.05 versus the corresponding negative control.

**Figure 2:**
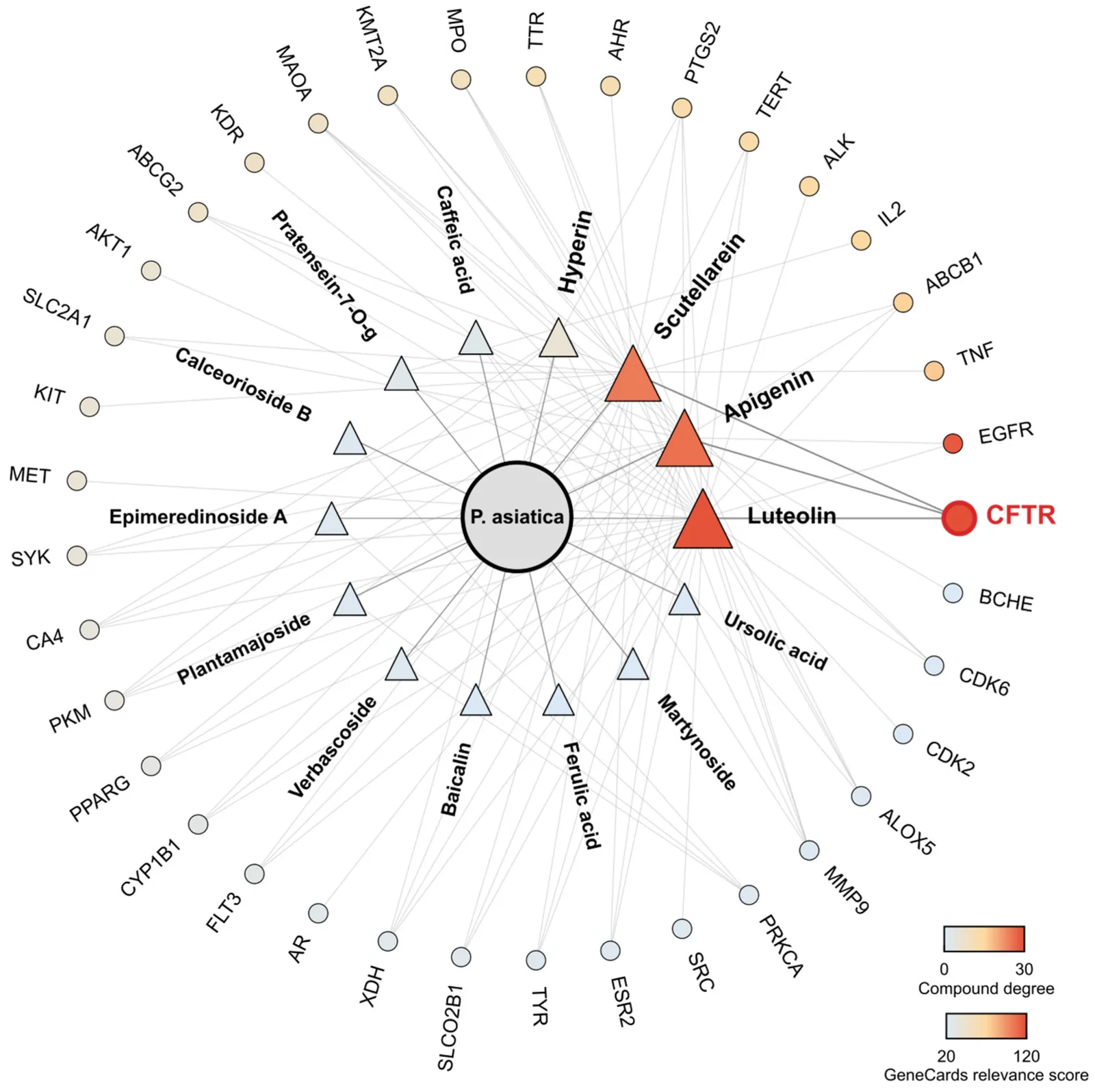
Compound–target network and phenotype-guided prioritisation of CFTR. Triangles denote the 14 active-fraction compounds and circles denote the 37 diarrhoea-associated targets retained after SwissTargetPrediction probability ≥ 0.9 and GeneCards relevance score ≥ 20 filtering. Compound colour/size indicates target degree; target colour indicates GeneCards relevance. CFTR ranked first and is highlighted in red.

**Figure 3:**
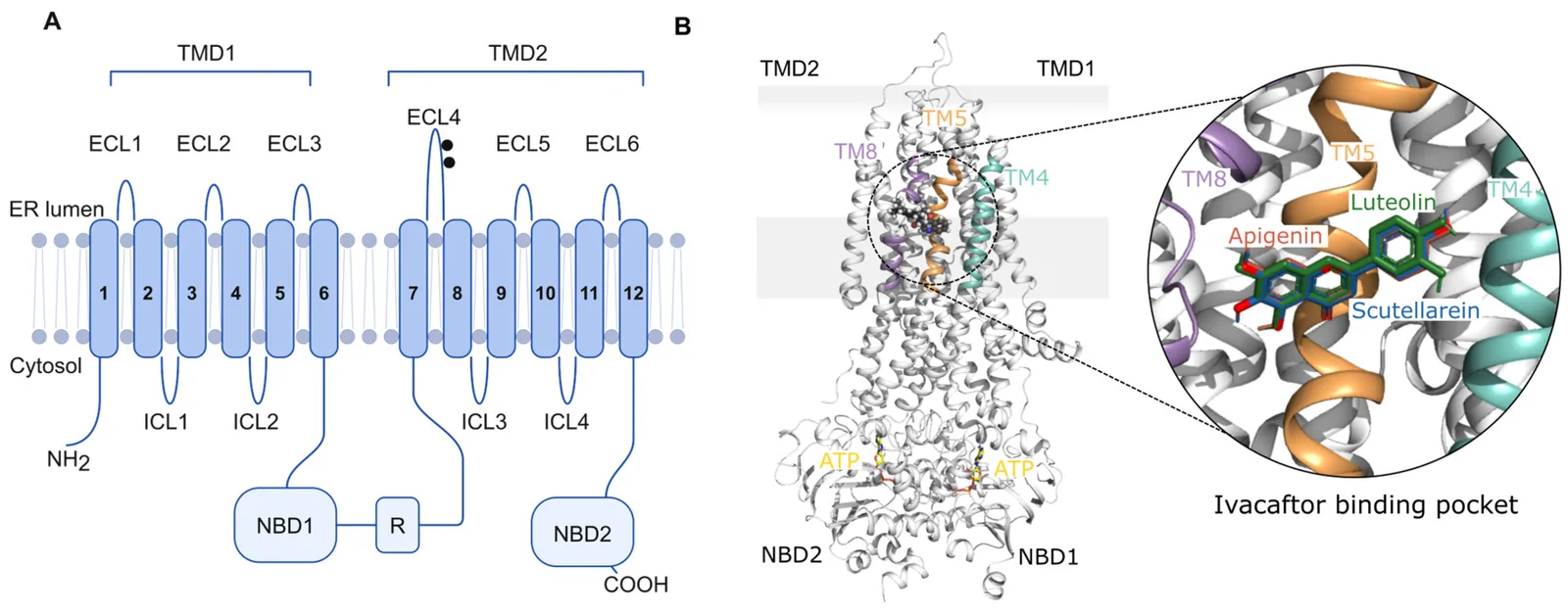
Structural location and starting poses at the ivacaftor-defined CFTR site. (**A**) Schematic domain organisation of full-length CFTR; unresolved loops and the R-domain segment were not rebuilt in the simulation model. (**B**) Membrane orientation of the porcine CFTR model and enlarged TM4/TM5/TM8 pocket showing structure-derived ivacaftor and the docked starting poses of apigenin, luteolin and scutellarein. Ivacaftor was retained from PDB 6O2P and was not re-docked.

**Figure 4:**
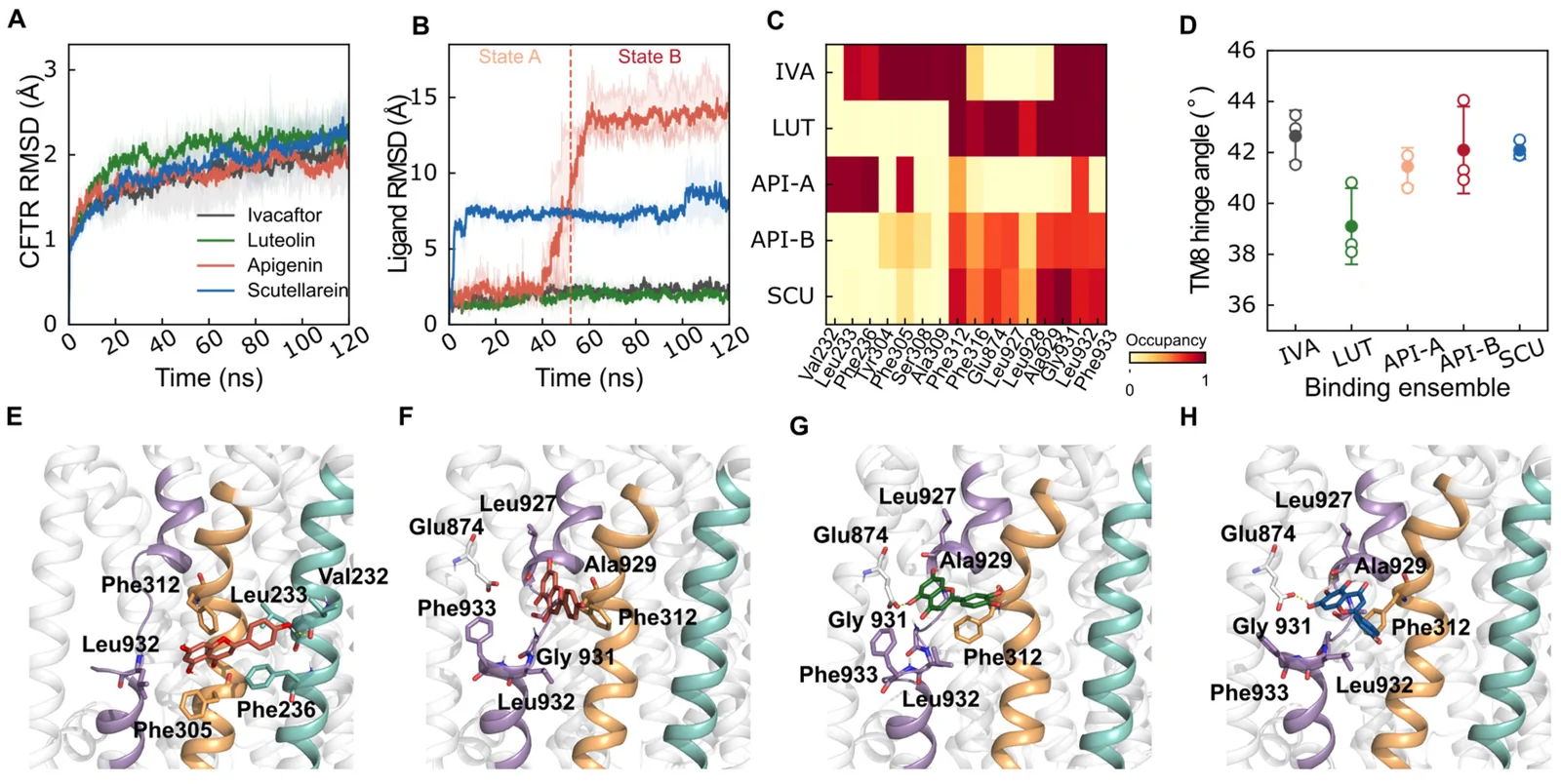
Stable and state-dependent flavonoid recognition at the lipid-exposed CFTR modulator site. (**A**) CFTR backbone RMSD; curves and bands show mean ± SD across three trajectories. (**B**) Ligand heavy-atom RMSD after protein fitting; the dashed line marks the median sustained apigenin transition, and labels indicate State A and State B. (**C**) State-resolved 4.5 Å contact occupancy for the 16 highest-scoring residues; complete values are provided in Table S15. (**D**) TM8 hinge-angle summaries; open circles are trajectory means, filled circles are ensemble means and bars show SD. (**E**) Apigenin TM4-shifted State A. (**F**) Apigenin TM8-facing State B. (**G**) Luteolin dominant ensemble. (**H**) Scutellarein dominant ensemble. TM4, TM5 and TM8 are cyan, orange and purple, respectively; residue labels use native porcine CFTR numbering. Dashed lines in (**E**–**H**) indicate hydrogen bonds present in the displayed representative frames.

**Figure 5:**
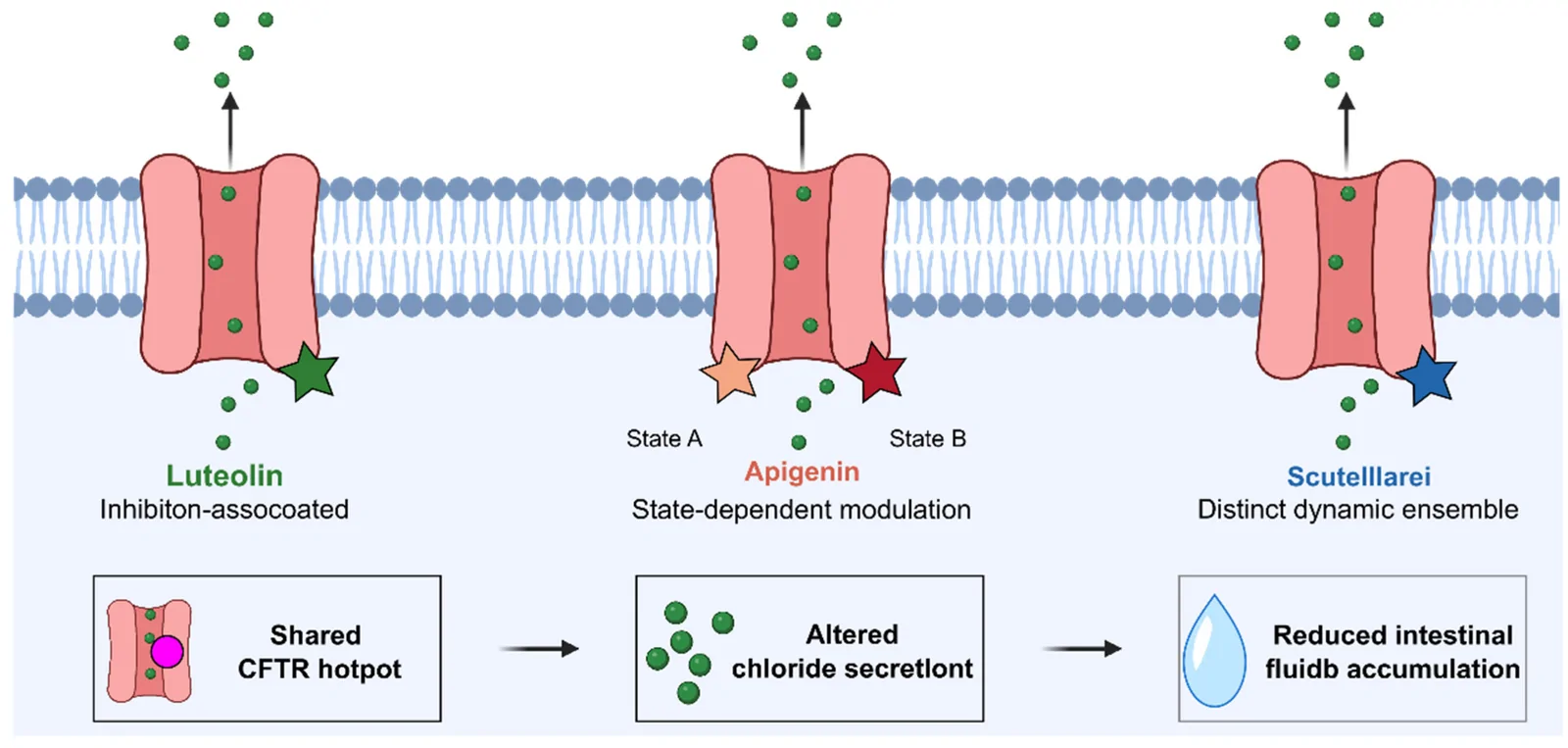
Proposed model of CFTR modulation by *P. asiatica* flavonoids. Luteolin is proposed to be associated with an inhibitory CFTR state, apigenin exhibits hypothesised state-dependent modulation, and scutellarein adopts a distinct dynamic binding ensemble. These ligand-specific effects at a shared CFTR hotspot may alter chloride secretion and reduce intestinal fluid accumulation. The proposed functional assignments represent structural hypotheses based on the simulated binding ensembles and published functional evidence.

**Table 1:** Compounds included in the active-fraction target-screening dataset.

Compound Code	Chemical Name	Structure	Classification
1	Hyperin	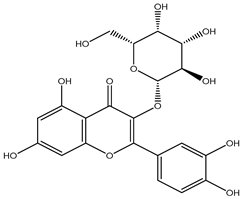	Flavonoid glycoside
2	Caffeic acid	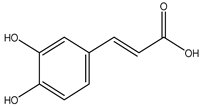	Phenolic acid
3	Martynoside	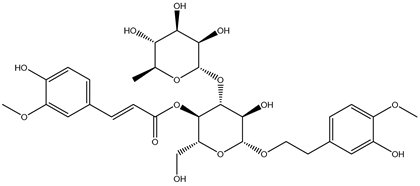	Phenylethanoid glycoside
4	Plantamajoside	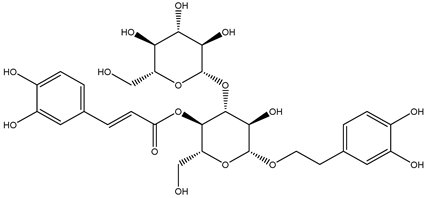	Phenylethanoid glycoside
5	Epimeredinoside A	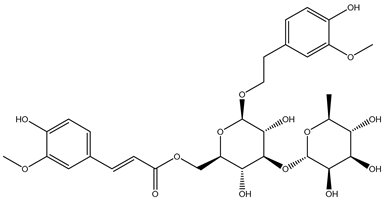	Phenylethanoid glycoside
6	Luteolin	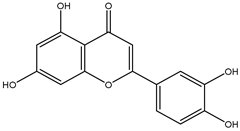	Flavonoid
7	Calceorioside B	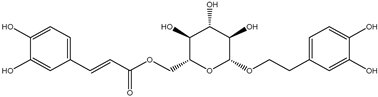	Phenylethanoid glycoside
8	Verbascoside	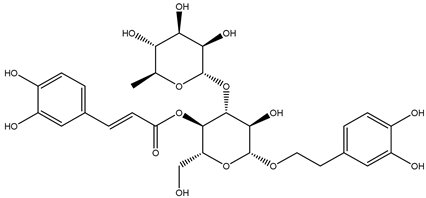	Phenylethanoid glycoside
9	Ursolic acid	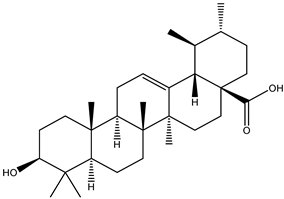	Triterpenoid
10	Apigenin	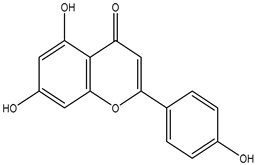	Flavonoid
11	Scutellarein	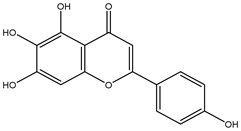	Flavonoid
12	Ferulic acid	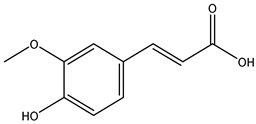	Phenolic acid
13	Pratensein-7-O-glucoside	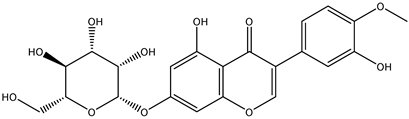	Flavonoid glycoside
14	Baicalin	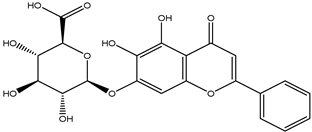	Flavonoid glycoside

## Data Availability

The numerical data underlying the figures, the molecular-dynamics analysis scripts and the full raw trajectories are available from the corresponding author on reasonable request. The animal data were originally recorded in Ren Y., MSc thesis, Northeast Agricultural University, 2017.

## Supplementary Materials

The following supporting information can be downloaded at: https://www.mdpi.com/article/10.3390/membranes16100308/s1. **Figure S1:** PROCHECK Ramachandran plot of the porcine CFTR model; **Figure S2:** ERRAT analysis of the porcine CFTR model; **Figure S3:** Equilibrated porcine CFTR membrane simulation systems; **Figure S4:** Apigenin state assignments across the three separately propagated trajectories; **Table S1:** Effects of the *Plantago asiatica* water extract in the castor-oil-induced diarrhoea model; **Table S2:** Effects of the water extract on charcoal-meal intestinal transit; **Table S3:** Effects of the water extract on intestinal fluid accumulation; **Table S4:** Effects of the partition fractions in the castor-oil-induced diarrhoea model; **Table S5:** Effects of the partition fractions on charcoal-meal intestinal transit; **Table S6:** Effects of the partition fractions on intestinal fluid accumulation; **Table S7A:** Predicted targets retained for the fourteen previously identified compounds; **Table S7B:** Thirty-seven disease-associated targets ranked by GeneCards relevance score; **Table S8:** Repeat-resolved ligand–protein hydrogen-bond occupancy; **Table S9:** Whole-trajectory ligand–CFTR heavy-atom contact occupancy; **Table S10:** Protein and ligand RMSD summaries for individual trajectories and across-trajectory means; **Table S11:** Membrane molecular-dynamics system composition and protocol; **Table S12:** Consecutive 40 ns protein and ligand RMSD block summaries; **Table S13A:** Sampled frame fractions of apigenin State A and State B in each trajectory; **Table S13B:** Sustained apigenin State B transition and post-transition persistence; **Table S14:** Simulation-topology to native porcine CFTR mapping for residues represented in the state-resolved contact analysis; **Table S15:** State-resolved ligand–CFTR heavy-atom contact occupancy; **Table S16:** State-resolved TM8 hinge-angle and TM5–TM8 pocket-distance summaries; **Table S17:** Atom counts and periodic-cell dimensions of the equilibrated membrane simulation systems.

## Abbreviations

AM1-BCC, Austin Model 1 bond charge correction; ATP, adenosine triphosphate; BuF, n-butanol fraction; CF, chloroform fraction; CFTR, cystic fibrosis transmembrane conductance regulator; EAF, ethyl acetate fraction; GAFF2, General Amber Force Field 2; MD, molecular dynamics; NBD, nucleotide-binding domain; NPT, constant number of particles, pressure and temperature ensemble; OPM, Orientations of Proteins in Membranes; PBC, periodic boundary conditions; PDB, Protein Data Bank; PEF, petroleum ether fraction; PME, particle mesh Ewald; POPC, 1-palmitoyl-2-oleoyl-sn-glycero-3-phosphocholine; RF, remaining aqueous fraction; RMSD, root-mean-square deviation; RMSF, root-mean-square fluctuation; UPLC-Q-TOF/MS, ultra-performance liquid chromatography coupled with quadrupole time-of-flight mass spectrometry.

## References

[1] Rhouma M., Fairbrother J.M., Beaudry F., Letellier A. (2017). Post weaning diarrhea in pigs: Risk factors and non-colistin-based control strategies. Acta Vet. Scand..

[2] Thiagarajah J.R., Donowitz M., Verkman A.S. (2015). Secretory diarrhoea: Mechanisms and emerging therapies. Nat. Rev. Gastroenterol. Hepatol..

[3] Keely S.J., Barrett K.E. (2022). Intestinal secretory mechanisms and diarrhea. Am. J. Physiol. Gastrointest. Liver Physiol..

[4] Field M. (2003). Intestinal ion transport and the pathophysiology of diarrhea. J. Clin. Investig..

[5] Barrett K.E., Keely S.J. (2000). Chloride secretion by the intestinal epithelium: Molecular basis and regulatory aspects. Annu. Rev. Physiol..

[6] Verkman A.S., Galietta L.J.V. (2009). Chloride channels as drug targets. Nat. Rev. Drug Discov..

[7] Ma T., Thiagarajah J.R., Yang H., Sonawane N.D., Folli C., Galietta L.J.V., Verkman A.S. (2002). Thiazolidinone CFTR inhibitor identified by high-throughput screening blocks cholera toxin-induced intestinal fluid secretion. J. Clin. Investig..

[8] Tradtrantip L., Namkung W., Verkman A.S. (2010). Crofelemer, an antisecretory antidiarrheal proanthocyanidin oligomer extracted from *Croton lechleri*, targets two distinct intestinal chloride channels. Mol. Pharmacol..

[9] Zhang Z., Liu F., Chen J. (2017). Conformational changes of CFTR upon phosphorylation and ATP binding. Cell.

[10] Csanády L., Vergani P., Gadsby D.C. (2019). Structure, gating, and regulation of the CFTR anion channel. Physiol. Rev..

[11] Hwang T.-C., Kirk K.L. (2013). The CFTR ion channel: Gating, regulation, and anion permeation. Cold Spring Harb. Perspect. Med..

[12] Liu F., Zhang Z., Levit A., Levring J., Touhara K.K., Shoichet B.K., Chen J. (2019). Structural identification of a hotspot on CFTR for potentiation. Science.

[13] Van Goor F., Hadida S., Grootenhuis P.D.J., Burton B., Stack J.H., Straley K.S., Decker C.J., Miller M., McCartney J., Olson E.R. (2009). Rescue of CF airway epithelial cell function in vitro by a CFTR potentiator, VX-770. Proc. Natl. Acad. Sci. USA.

[14] Jih K.-Y., Hwang T.-C. (2013). VX-770 potentiates CFTR function by promoting decoupling between the gating cycle and ATP hydrolysis cycle. Proc. Natl. Acad. Sci. USA.

[15] Liu F., Kaplan A.L., Levring J., Einsiedel J., Tiedt S., Distler K., Omattage N.S., Kondratov I.S., Moroz Y.S., Pietz H.L. (2024). Structure-based discovery of CFTR potentiators and inhibitors. Cell.

[16] Chinese Pharmacopoeia Commission (2020). Pharmacopoeia of the People’s Republic of China.

[17] Zhang S., Hu J., Sun Y., Tan H., Yin J., Geng F., Nie S. (2021). Review of structure and bioactivity of the *Plantago* (Plantaginaceae) polysaccharides. Food Chem. X.

[18] Hu R., Lin C., Xu W., Liu Y., Long C. (2020). Ethnobotanical study on medicinal plants used by Mulam people in Guangxi, China. J. Ethnobiol. Ethnomed..

[19] Dong C.-L., Qin Y., Ma J.-X., Cui W.-Q., Chen X.-R., Hou L.-Y., Chen X.-Y., Bello-Onaghise G., Eliphaz N., Qin J.-J. (2021). The active ingredients identification and antidiarrheal mechanism analysis of *Plantago asiatica* L. superfine powder. Front. Pharmacol..

[20] Wen S.-Y., Wei B.-Y., Ma J.-Q., Wang L., Chen Y.-Y. (2023). Phytochemicals, biological activities, molecular mechanisms, and future prospects of *Plantago asiatica* L.. J. Agric. Food Chem..

[21] Daina A., Michielin O., Zoete V. (2019). SwissTargetPrediction: Updated data and new features for efficient prediction of protein targets of small molecules. Nucleic Acids Res..

[22] Safran M., Dalah I., Alexander J., Rosen N., Iny Stein T., Shmoish M., Nativ N., Bahir I., Doniger T., Krug H. (2010). GeneCards Version 3: The human gene integrator. Database.

[23] Illek B., Lizarzaburu M.E., Lee V., Nantz M.H., Kurth M.J., Fischer H. (2000). Structural determinants for activation and block of CFTR-mediated chloride currents by apigenin. Am. J. Physiol. Cell Physiol..

[24] Schuier M., Sies H., Illek B., Fischer H. (2005). Cocoa-related flavonoids inhibit CFTR-mediated chloride transport across T84 human colon epithelia. J. Nutr..

[25] Gao X., Li S., Cong C., Wang Y., Xu L. (2021). A network pharmacology approach to estimate potential targets of the active ingredients of *Epimedium* for alleviating mild cognitive impairment and treating Alzheimer’s disease. Evid. Based Complement. Altern. Med..

[26] Ostedgaard L.S., Rogers C.S., Dong Q., Randak C.O., Vermeer D.W., Rokhlina T., Karp P.H., Welsh M.J. (2007). Processing and function of CFTR-ΔF508 are species-dependent. Proc. Natl. Acad. Sci. USA.

[27] Laskowski R.A., MacArthur M.W., Moss D.S., Thornton J.M. (1993). PROCHECK: A program to check the stereochemical quality of protein structures. J. Appl. Crystallogr..

[28] Colovos C., Yeates T.O. (1993). Verification of protein structures: Patterns of nonbonded atomic interactions. Protein Sci..

[29] Friesner R.A., Banks J.L., Murphy R.B., Halgren T.A., Klicic J.J., Mainz D.T., Repasky M.P., Knoll E.H., Shelley M., Perry J.K. (2004). Glide: A new approach for rapid, accurate docking and scoring. 1. Method and assessment of docking accuracy. J. Med. Chem..

[30] Lomize M.A., Pogozheva I.D., Joo H., Mosberg H.I., Lomize A.L. (2012). OPM database and PPM web server: Resources for positioning of proteins in membranes. Nucleic Acids Res..

[31] Schott-Verdugo S., Gohlke H. (2019). PACKMOL-Memgen: A simple-to-use, generalized workflow for membrane-protein-lipid-bilayer system building. J. Chem. Inf. Model..

[32] Martínez L., Andrade R., Birgin E.G., Martínez J.M. (2009). PACKMOL: A package for building initial configurations for molecular dynamics simulations. J. Comput. Chem..

[33] Tian C., Kasavajhala K., Belfon K.A.A., Raguette L., Huang H., Migues A.N., Bickel J., Wang Y., Pincay J., Wu Q. (2020). ff19SB: Amino-acid-specific protein backbone parameters trained against quantum mechanics energy surfaces in solution. J. Chem. Theory Comput..

[34] Dickson C.J., Walker R.C., Gould I.R. (2022). Lipid21: Complex lipid membrane simulations with AMBER. J. Chem. Theory Comput..

[35] Wang J., Wolf R.M., Caldwell J.W., Kollman P.A., Case D.A. (2004). Development and testing of a general Amber force field. J. Comput. Chem..

[36] Jakalian A., Jack D.B., Bayly C.I. (2002). Fast, efficient generation of high-quality atomic charges. II. Parameterization and validation of the AM1-BCC model. J. Comput. Chem..

[37] Izadi S., Anandakrishnan R., Onufriev A.V. (2014). Building water models: A different approach. J. Phys. Chem. Lett..

[38] Meagher K.L., Redman L.T., Carlson H.A. (2003). Development of polyphosphate parameters for use with the AMBER force field. J. Comput. Chem..

[39] Case D.A., Aktulga H.M., Belfon K., Ben-Shalom I.Y., Berryman J.T., Brozell S.R., Carvalho F.S., Cerutti D.S., Cheatham T.E., Cisneros G.A. (2025). Amber 2025 Reference Manual (Covers Amber24 and AmberTools25).

[40] Roe D.R., Cheatham T.E. (2013). PTRAJ and CPPTRAJ: Software for processing and analysis of molecular dynamics trajectory data. J. Chem. Theory Comput..

[41] Michaud-Agrawal N., Denning E.J., Woolf T.B., Beckstein O. (2011). MDAnalysis: A toolkit for the analysis of molecular dynamics simulations. J. Comput. Chem..

[42] Gowers R.J., Linke M., Barnoud J., Reddy T.J.E., Melo M.N., Seyler S.L., Domański J., Dotson D.L., Buchoux S., Kenney I.M. MDAnalysis: A Python package for the rapid analysis of molecular dynamics simulations. Proceedings of the 15th Python in Science Conference.

[43] Dror R.O., Dirks R.M., Grossman J.P., Xu H., Shaw D.E. (2012). Biomolecular simulation: A computational microscope for molecular biology. Annu. Rev. Biophys..

[44] Knapp B., Ospina L., Deane C.M. (2018). Avoiding false positive conclusions in molecular simulation: The importance of replicas. J. Chem. Theory Comput..

