# Supplementary material for: CFTR Modulation by Plantago asiatica Flavonoids: A Potential Antisecretory Mechanism Against Diarrhoea
Source: Membranes (Basel). 2026 Sep 22;16(10):308. doi: 10.3390/membranes16100308 (PMC13707013; doi:10.3390/membranes16100308)
Supplement: Supplementary file 1 [file membranes-16-00308-s001.zip › membranes-4529531-supplementary.pdf]

Supplementary Information

CFTR Modulation by *Plantago asiatica* Flavonoids: A Potential Antisecretory Mechanism against Diarrhoea

Wenqiang Cui[1,2], JiaHao Liu[1,2], Ruiguang Wang[1,2], Yongzhi Ren[1,2] and Yanhua Li[1,2, *]

[1] College of Veterinary Medicine, Northeast Agricultural University, Harbin 150030, China

[2] Heilongjiang Key Laboratory for Animal Disease Control and Pharmaceutical Development, Harbin 150030, China

*** Correspondence:**

Yanhua Li: liyanhua@neau.edu.cn

Table S1. Effects of the *Plantago asiatica* water extract in the castor-oil-induced diarrhoea model.

Experiment	Treatment	Dose (mg/kg)	n	Onset (min)	Wet faeces (n/6 h)	Protection (%)	Significant vs. control
Water extract	Negative control	—	6	65.60 ± 4.15	10.70 ± 1.02	—	—
Water extract	Water extract	150	6	71.40 ± 7.32	7.30 ± 0.98	31.78	Yes
Water extract	Water extract	300	6	74.20 ± 9.68	6.50 ± 0.56	39.25	Yes
Water extract	Water extract	450	6	81.10 ± 8.53	5.60 ± 0.15	47.66	Yes
Water extract	Loperamide	2	6	154.00 ± 12.64	5.20 ± 0.31	51.40	Yes

Table S2. Effects of the water extract on charcoal-meal intestinal transit.

Experiment	Treatment	Dose (mg/kg)	n	Intestinal length (cm)	Charcoal distance (cm)	Transit (%)	Inhibition (%)	Significant vs. control
Water extract	Negative control	—	6	51.25 ± 2.14	36.89 ± 2.69	71.98 ± 2.68	—	—
Water extract	Water extract	150	6	49.86 ± 1.89	34.56 ± 2.69	69.31 ± 1.87	3.71	No
Water extract	Water extract	300	6	50.62 ± 1.96	33.54 ± 2.81	66.26 ± 2.57	7.95	No
Water extract	Water extract	450	6	50.35 ± 3.62	31.43 ± 1.52	62.42 ± 1.72	13.28	No
Water extract	Atropine sulfate	2	6	50.43 ± 2.65	17.53 ± 1.32	34.76 ± 1.91	51.71	Yes

Table S3. Effects of the water extract on intestinal fluid accumulation.

Experiment	Treatment	Dose (mg/kg)	n	Fluid volume (mL)	Content weight (g)	Inhibition (%)	Significant vs. control
Water extract	Negative control	—	6	3.24 ± 0.02	3.51 ± 0.02	—	—
Water extract	Water extract	150	6	2.01 ± 0.06	2.88 ± 0.01	37.96	Yes
Water extract	Water extract	300	6	1.89 ± 0.05	2.65 ± 0.03	41.67	Yes
Water extract	Water extract	450	6	1.52 ± 0.02	2.49 ± 0.03	53.09	Yes
Water extract	Atropine sulfate	2	6	1.39 ± 0.01	1.68 ± 0.02	57.10	Yes

Table S4. Effects of the partition fractions in the castor-oil-induced diarrhoea model.

Experiment	Treatment	Dose (mg/kg)	n	Onset (min)	Wet faeces (n/6 h)	Protection (%)	Significant vs. control
PEF	Negative control	—	6	74.36 ± 6.54	10.20 ± 0.68	—	—

PEF	PEF	150	6	69.89 ± 5.39	11.02 ± 1.25	-8.04	No
PEF	PEF	300	6	72.55 ± 8.67	9.80 ± 0.96	3.92	No
PEF	PEF	450	6	71.54 ± 11.24	10.50 ± 0.59	-2.94	No
PEF	Loperamide	2	6	159.00 ± 13.27	4.90 ± 0.19	51.96	Yes
CF	Negative control	—	6	81.54 ± 10.47	9.80 ± 0.87	—	—
CF	CF	150	6	69.61 ± 11.69	10.70 ± 1.11	-9.18	No
CF	CF	300	6	61.57 ± 10.78	9.70 ± 0.54	1.02	No
CF	CF	450	6	67.39 ± 12.92	10.40 ± 0.62	-6.12	No
CF	Loperamide	2	6	172.24 ± 18.64	4.80 ± 0.26	51.02	Yes
EAF	Negative control	—	6	76.60 ± 9.87	11.40 ± 1.25	—	—
EAF	EAF	150	6	98.86 ± 10.24	6.40 ± 1.03	43.86	Yes
EAF	EAF	300	6	116.46 ± 13.24	5.20 ± 0.62	54.39	Yes
EAF	EAF	450	6	168.64 ± 17.62	3.50 ± 0.21	69.30	Yes
EAF	Loperamide	2	6	158.62 ± 12.63	4.80 ± 0.34	57.89	Yes
BuF	Negative control	—	6	62.70 ± 7.54	9.65 ± 0.86	—	—
BuF	BuF	150	6	52.30 ± 6.24	11.25 ± 1.28	-16.58	No
BuF	BuF	300	6	57.60 ± 7.98	10.68 ± 0.39	-10.67	No
BuF	BuF	450	6	49.40 ± 4.13	12.46 ± 0.89	-29.12	No
BuF	Loperamide	2	6	149.50 ± 12.56	4.58 ± 0.41	52.54	Yes
RF	Negative control	—	6	67.54 ± 8.26	10.58 ± 0.86	—	—
RF	RF	150	6	87.62 ± 11.52	6.90 ± 0.92	34.78	Yes
RF	RF	300	6	106.28 ± 16.32	5.70 ± 0.25	46.12	Yes
RF	RF	450	6	132.51 ± 10.82	4.70 ± 0.36	55.58	Yes
RF	Loperamide	2	6	143.85 ± 19.10	4.50 ± 0.18	57.47	Yes

Table S5. Effects of the partition fractions on charcoal-meal intestinal transit.

Experiment	Treatment	Dose (mg/kg)	n	Intestinal length (cm)	Charcoal distance (cm)	Transit (%)	Inhibition (%)	Significant vs. control
PEF	Negative	—	6	50.64 ±	36.27 ±	71.62	—	—

	control			2.45	1.56	± 2.89		
PEF	PEF	150	6	51.41 ± 3.26	37.84 ± 2.54	73.60 ± 3.21	-2.76	No
PEF	PEF	300	6	49.64 ± 1.82	34.86 ± 1.57	70.23 ± 1.42	1.94	No
PEF	PEF	450	6	50.34 ± 1.74	35.98 ± 1.76	71.47 ± 3.26	0.21	No
PEF	Atropine sulfate	2	6	51.26 ± 1.63	17.54 ± 1.48	34.21 ± 1.76	52.23	Yes
CF	Negative control	—	6	46.37 ± 2.31	32.57 ± 1.97	70.24 ± 3.51	—	—
CF	CF	150	6	48.31 ± 3.06	35.53 ± 2.48	73.54 ± 1.64	-4.70	No
CF	CF	300	6	44.26 ± 1.32	34.11 ± 2.04	77.07 ± 2.14	-9.72	No
CF	CF	450	6	47.25 ± 3.43	35.14 ± 1.63	74.37 ± 1.33	-5.88	No
CF	Atropine sulfate	2	6	48.10 ± 3.51	15.98 ± 0.92	33.22 ± 2.24	52.71	Yes
EAF	Negative control	—	6	54.62 ± 4.25	37.48 ± 3.68	68.62 ± 2.51	—	—
EAF	EAF	150	6	52.36 ± 2.81	21.42 ± 1.65	40.91 ± 1.53	40.38	Yes
EAF	EAF	300	6	53.28 ± 3.26	16.81 ± 1.82	31.55 ± 1.52	54.02	Yes
EAF	EAF	450	6	55.21 ± 4.62	14.49 ± 1.12	26.25 ± 1.21	61.75	Yes
EAF	Atropine sulfate	2	6	52.81 ± 1.82	15.82 ± 1.91	29.96 ± 1.35	56.34	Yes
BuF	Negative control	—	6	45.89 ± 2.69	30.69 ± 3.03	66.88 ± 2.13	—	—
BuF	BuF	150	6	49.64 ± 1.09	34.19 ± 1.23	68.88 ± 2.80	-2.99	No
BuF	BuF	300	6	46.15 ± 1.96	32.38 ± 1.69	70.16 ± 2.82	-4.90	No
BuF	BuF	450	6	44.64 ± 0.89	32.58 ± 3.61	72.98 ± 2.09	-9.12	No
BuF	Atropine sulfate	2	6	46.60 ± 3.08	14.98 ± 0.57	32.15 ± 1.36	51.93	Yes
RF	Negative control	—	6	48.62 ± 3.93	33.52 ± 2.19	68.94 ± 3.05	—	—
RF	RF	150	6	49.31 ± 2.69	22.39 ± 1.29	45.41 ± 1.89	34.13	Yes
RF	RF	300	6	46.29 ± 3.26	18.65 ± 1.08	40.29 ± 1.58	41.56	Yes
RF	RF	450	6	48.93 ± 4.08	15.36 ± 1.33	31.39 ± 0.88	54.47	Yes
RF	Atropine sulfate	2	6	47.92 ± 1.63	14.68 ± 0.98	30.63 ± 1.16	55.57	Yes

Table S6. Effects of the partition fractions on intestinal fluid accumulation.

Experiment	Treatment	Dose (mg/kg)	n	Fluid volume (mL)	Content weight (g)	Inhibition (%)	Significant vs. control
PEF	Negative	—	6	3.31 ±	3.66 ±	—	—

	control			0.03	0.05		
PEF	PEF	150	6	3.35 ± 0.02	3.70 ± 0.04	-1.21	No
PEF	PEF	300	6	3.28 ± 0.04	3.54 ± 0.02	0.91	No
PEF	PEF	450	6	3.30 ± 0.02	3.62 ± 0.01	0.30	No
PEF	Atropine sulfate	2	6	1.48 ± 0.01	1.65 ± 0.02	55.29	Yes
CF	Negative control	—	6	2.86 ± 0.01	3.04 ± 0.02	—	—
CF	CF	150	6	2.94 ± 0.05	3.19 ± 0.06	-2.80	No
CF	CF	300	6	2.76 ± 0.04	2.98 ± 0.04	3.50	No
CF	CF	450	6	2.84 ± 0.03	3.07 ± 0.01	0.70	No
CF	Atropine sulfate	2	6	1.21 ± 0.02	1.59 ± 0.02	57.69	Yes
EAF	Negative control	—	6	3.35 ± 0.02	3.57 ± 0.03	—	—
EAF	EAF	150	6	1.91 ± 0.02	2.26 ± 0.01	42.99	Yes
EAF	EAF	300	6	1.59 ± 0.03	1.89 ± 0.02	52.54	Yes
EAF	EAF	450	6	1.23 ± 0.01	1.63 ± 0.01	63.28	Yes
EAF	Atropine sulfate	2	6	1.42 ± 0.02	1.74 ± 0.02	57.61	Yes
BuF	Negative control	—	6	2.89 ± 0.01	3.23 ± 0.02	—	—
BuF	BuF	150	6	2.85 ± 0.02	3.19 ± 0.01	1.38	No
BuF	BuF	300	6	2.96 ± 0.03	3.39 ± 0.02	-2.42	No
BuF	BuF	450	6	3.02 ± 0.04	3.41 ± 0.02	-4.50	No
BuF	Atropine sulfate	2	6	1.29 ± 0.01	1.61 ± 0.01	55.36	Yes
RF	Negative control	—	6	3.19 ± 0.01	3.38 ± 0.02	—	—
RF	RF	150	6	2.14 ± 0.02	2.48 ± 0.01	32.92	Yes
RF	RF	300	6	1.92 ± 0.01	2.31 ± 0.02	39.81	Yes
RF	RF	450	6	1.69 ± 0.02	2.05 ± 0.01	47.02	Yes
RF	Atropine sulfate	2	6	1.56 ± 0.02	1.98 ± 0.01	51.10	Yes

Table S7A. Predicted targets retained for the fourteen previously identified compounds.

compound	target_full_name	gene_symbol	uniprot_id	target_class	probability	known_actives_3D_2D	genecards_relevance_score

Apigenin	Broad substrate specificity ATP-binding cassette transporter ABCG2	ABCG2	Q9UNQ0	Primary active transporter	1.000	39 / 153	46.25
Apigenin	Estrogen receptor beta	ESR2	Q92731	Nuclear receptor	1.000	586 / 129	24.44
Apigenin	Cytochrome P450 1B1	CYP1B1	Q16678	Cytochrome P450	1.000	34 / 152	31.16
Apigenin	Cyclin-dependent kinase 6	CDK6	Q00534	Kinase	1.000	9 / 6	21.75
Apigenin	Xanthine dehydrogenase/oxidase	XDH	P47989	Oxidoreductase	1.000	147 / 47	26.26
Apigenin	Tyrosine-protein kinase SYK	SYK	P43405	Kinase	1.000	26 / 3	38.16
Apigenin	Receptor-type tyrosine-protein kinase FLT3	FLT3	P36888	Kinase	1.000	52 / 17	29.46
Apigenin	Prostaglandin G/H synthase 2	PTGS2	P35354	Oxidoreductase	1.000	108 / 111	62.74
Apigenin	Amine oxidase [flavin-containing] A	MAOA	P21397	Oxidoreductase	1.000	151 / 126	47.96
Apigenin	Pyruvate kinase PKM	PKM	P14618	Enzyme	1.000	17 / 9	34.41
Apigenin	Cystic fibrosis transmembrane conductance regulator	CFTR	P13569	Other ion channel	1.000	7 / 3	117.84
Apigenin	Myeloperoxidase	MPO	P05164	Oxidoreductase	1.000	20 / 4	52.30
Apigenin	Transthyretin	TTR	P02766	Secreted protein	1.000	53 / 9	57.82
Apigenin	Solute carrier organic anion transporter family member 2B1	SLCO2B1	O94956	Electrochemical transporter	0.990	7 / 6	25.49

Apigenin	Polyunsaturated fatty acid 5-lipoxygenase	ALOX5	P09917	Oxidoreductase	0.978	200 / 79	23.14
Apigenin	Peroxisome proliferator-activated receptor gamma	PPARG	P37231	Nuclear receptor	0.976	32 / 16	32.65
Apigenin	Carbonic anhydrase 4	CA4	P22748	Lyase	0.976	51 / 35	35.35
Apigenin	ATP-dependent translocase ABCB1	ABCB1	P08183	Primary active transporter	0.976	47 / 114	72.66
Apigenin	Solute carrier family 2, facilitated glucose transporter member 1	SLC2A1	P11166	Electrochemical transporter	0.966	3 / 2	40.84
Apigenin	Matrix metalloproteinase-9	MMP9	P14780	Protease	0.962	73 / 6	23.65
Apigenin	Tyrosinase	TYR	P14679	Oxidoreductase	0.956	107 / 34	24.98
Apigenin	Histone-lysine N-methyltransferase 2A	KMT2A	Q03164	Writer	0.955	1 / 1	50.55
Apigenin	Telomerase reverse transcriptase	TERT	O14746	Transferase	0.954	31 / 27	63.70
Apigenin	Androgen receptor	AR	P10275	Nuclear receptor	0.951	155 / 38	26.59
Apigenin	Cholinesterase	BCHE	P06276	Hydrolase	0.918	37 / 95	20.43
Apigenin	Epidermal growth factor receptor	EGFR	P00533	Kinase	0.917	178 / 39	114.60
Baicalin	-	-	-	-	-	-	-
Caffeic acid	Matrix metalloproteinase-9	MMP9	P14780	Protease	1.000	42 / 76	23.65
Caffeic acid	Polyunsaturated fatty acid 5-lipoxygenase	ALOX5	P09917	Oxidoreductase	1.000	96 / 131	23.14
Calceori	Protein	PRKC	P17	Kinase	1.00	128 / 315	23.67

oside B	kinase C alpha type	A	252		0		
Epimeredinoside A	Protein kinase C alpha type	PRKCA	P17252	Kinase	0.943	144 / 304	23.67
Ferulic acid	-	-	-	-	-	-	-
Hyperin	Xanthine dehydrogenase/oxidase	XDH	P47989	Oxidoreductase	0.998	172 / 28	26.26
Hyperin	Carbonic anhydrase 4	CA4	P22748	Lyase	0.998	77 / 12	35.35
Hyperin	Pyruvate kinase PKM	PKM	P14618	Enzyme	0.998	20 / 10	34.41
Hyperin	Matrix metalloproteinase-9	MMP9	P14780	Protease	0.996	129 / 5	23.65
Hyperin	Amine oxidase [flavin-containing] A	MAOA	P21397	Oxidoreductase	0.984	222 / 22	47.96
Hyperin	Prostaglandin G/H synthase 2	PTGS2	P35354	Oxidoreductase	0.973	154 / 72	62.74
Luteolin	Polyunsaturated fatty acid 5-lipoxygenase	ALOX5	P09917	Oxidoreductase	1.000	84 / 82	23.14
Luteolin	Solute carrier organic anion transporter family member 2B1	SLCO2B1	O94956	Electrochemical transporter	1.000	7 / 6	25.49
Luteolin	Xanthine dehydrogenase/oxidase	XDH	P47989	Oxidoreductase	1.000	65 / 53	26.26
Luteolin	Amine oxidase [flavin-containing] A	MAOA	P21397	Oxidoreductase	1.000	62 / 112	47.96
Luteolin	Matrix metalloproteinase-9	MMP9	P14780	Protease	1.000	43 / 6	23.65
Luteolin	Carbonic anhydrase 4	CA4	P22748	Lyase	1.000	42 / 31	35.35
Luteolin	Transthyretin	TTR	P02766	Secreted protein	1.000	36 / 9	57.82

Luteolin	Receptor-type tyrosine-protein kinase FLT3	FLT3	P36888	Kinase	1.000	27 / 16	29.46
Luteolin	Cytochrome P450 1B1	CYP1B1	Q16678	Cytochrome P450	1.000	25 / 151	31.16
Luteolin	Broad substrate specificity ATP-binding cassette transporter ABCG2	ABCG2	Q9UNQ0	Primary active transporter	1.000	20 / 163	46.25
Luteolin	Tyrosine-protein kinase SYK	SYK	P43405	Kinase	1.000	10 / 3	38.16
Luteolin	Cyclin-dependent kinase 6	CDK6	Q00534	Kinase	0.983	6 / 6	21.75
Luteolin	Pyruvate kinase PKM	PKM	P14618	Enzyme	0.983	13 / 9	34.41
Luteolin	Myeloperoxidase	MPO	P05164	Oxidoreductase	0.978	8 / 4	52.30
Luteolin	Telomerase reverse transcriptase	TERT	O14746	Transferase	0.976	22 / 27	63.70
Luteolin	Histone-lysine N-methyltransferase 2A	KMT2A	Q03164	Writer	0.976	1 / 1	50.55
Luteolin	Estrogen receptor beta	ESR2	Q92731	Nuclear receptor	0.968	273 / 127	24.44
Luteolin	ATP-dependent translocase ABCB1	ABCB1	P08183	Primary active transporter	0.963	23 / 123	72.66
Luteolin	Tyrosinase	TYR	P14679	Oxidoreductase	0.949	64 / 33	24.98
Luteolin	ALK tyrosine kinase receptor	ALK	Q9UM73	Kinase	0.949	9 / 4	66.24
Luteolin	Hepatocyte growth factor receptor	MET	P08581	Kinase	0.949	81 / 5	40.15
Luteolin	Vascular endothelial growth factor receptor 2	KDR	P35968	Kinase	0.949	31 / 11	47.15
Luteolin	Cyclin-	CDK2	P24	Kinase	0.94	49 / 26	22.00

	dependent kinase 2		941		6		
Luteolin	Peroxisome proliferator-activated receptor gamma	PPARG	P37231	Nuclear receptor	0.941	11 / 16	32.65
Luteolin	Prostaglandin G/H synthase 2	PTGS2	P35354	Oxidoreductase	0.941	54 / 108	62.74
Luteolin	RAC-alpha serine/threonine-protein kinase	AKT1	P31749	Kinase	0.926	8 / 5	41.53
Luteolin	Proto-oncogene tyrosine-protein kinase Src	SRC	P12931	Kinase	0.926	67 / 11	24.32
Luteolin	Cystic fibrosis transmembrane conductance regulator	CFTR	P13569	Other ion channel	0.925	3 / 3	117.84
Luteolin	Epidermal growth factor receptor	EGFR	P00533	Kinase	0.909	100 / 39	114.60
Martynoside	-	-	-	-	-	-	-
Plantamajoside	Protein kinase C alpha type	PRKCA	P17252	Kinase	0.995	143 / 317	23.67
Pratensein-7-O-glucoside	Tumor necrosis factor	TNF	P01375	Secreted protein	0.993	47 / 3	75.60
Pratensein-7-O-glucoside	Interleukin-2	IL2	P60568	Secreted protein	0.982	23 / 5	70.17
Scutellarein	Histone-lysine N-methyltransferase 2A	KMT2A	Q03164	Writer	1.000	1 / 1	50.55
Scutellarein	Solute carrier organic anion transporter family member 2B1	SLCO2B1	O94956	Electrochemical transporter	1.000	7 / 6	25.49
Scutellarein	Broad substrate specificity	ABCG2	Q9UNQ0	Primary active transpo	0.988	26 / 156	46.25

	ATP-binding cassette transporter ABCG2			rter			
Scutellarein	Cytochrome P450 1B1	CYP1B1	Q16678	Cytochrome P450	0.988	26 / 152	31.16
Scutellarein	Xanthine dehydrogenase/oxidase	XDH	P47989	Oxidoreductase	0.988	76 / 45	26.26
Scutellarein	Tyrosine-protein kinase SYK	SYK	P43405	Kinase	0.988	14 / 3	38.16
Scutellarein	Receptor-type tyrosine-protein kinase FLT3	FLT3	P36888	Kinase	0.988	30 / 16	29.46
Scutellarein	Amine oxidase [flavin-containing] A	MAOA	P21397	Oxidoreductase	0.988	76 / 116	47.96
Scutellarein	Transthyretin	TTR	P02766	Secreted protein	0.988	41 / 9	57.82
Scutellarein	Myeloperoxidase	MPO	P05164	Oxidoreductase	0.987	11 / 4	52.30
Scutellarein	Cyclin-dependent kinase 6	CDK6	Q00534	Kinase	0.982	8 / 6	21.75
Scutellarein	Pyruvate kinase PKM	PKM	P14618	Enzyme	0.982	15 / 9	34.41
Scutellarein	Carbonic anhydrase 4	CA4	P22748	Lyase	0.978	41 / 29	35.35
Scutellarein	Estrogen receptor beta	ESR2	Q92731	Nuclear receptor	0.978	314 / 129	24.44
Scutellarein	ATP-dependent translocase ABCB1	ABCB1	P08183	Primary active transporter	0.977	30 / 117	72.66
Scutellarein	Matrix metalloproteinase-9	MMP9	P14780	Protease	0.976	43 / 6	23.65
Scutellarein	Polyunsaturated fatty acid 5-lipoxygenase	ALOX5	P09917	Oxidoreductase	0.976	95 / 79	23.14
Scutellarein	Cystic fibrosis transmembrane conductanc	CFTR	P13569	Other ion channel	0.964	5 / 3	117.84

	e regulator						
Scutellarein	Prostaglandin G/H synthase 2	PTGS2	P35354	Oxidoreductase	0.961	60 / 112	62.74
Scutellarein	Tyrosinase	TYR	P14679	Oxidoreductase	0.957	72 / 33	24.98
Scutellarein	Peroxisome proliferator-activated receptor gamma	PPARG	P37231	Nuclear receptor	0.956	11 / 16	32.65
Scutellarein	Aryl hydrocarbon receptor	AHR	P35869	Transcription factor	0.945	8 / 4	58.96
Scutellarein	Solute carrier family 2, facilitated glucose transporter member 1	SLC2A1	P11166	Electrochemical transporter	0.945	2 / 2	40.84
Scutellarein	Telomerase reverse transcriptase	TERT	O14746	Transferase	0.941	24 / 27	63.70
Scutellarein	Mast/stem cell growth factor receptor Kit	KIT	P10721	Kinase	0.920	8 / 3	40.81
Ursolic acid	-	-	-	-	-	-	-
Verbascoside	Protein kinase C alpha type	PRKCA	P17252	Kinase	1.000	138 / 317	23.67

Table S7B. Thirty-seven disease-associated targets ranked by GeneCards relevance score.

rank	gene_symbol	genecards_relevance_score	n_compounds_hitting	compounds
1	CFTR	117.84	3	Apigenin; Luteolin; Scutellarein
2	EGFR	114.60	2	Apigenin; Luteolin
3	TNF	75.60	1	Pratensein-7-O-glucoside
4	ABCB1	72.66	3	Apigenin; Luteolin; Scutellarein
5	IL2	70.17	1	Pratensein-7-O-glucoside
6	ALK	66.24	1	Luteolin
7	TERT	63.70	3	Apigenin; Luteolin; Scutellarein

8	PTGS2	62.74	4	Apigenin; Hyperin; Luteolin; Scutellarein
9	AHR	58.96	1	Scutellarein
10	TTR	57.82	3	Apigenin; Luteolin; Scutellarein
11	MPO	52.30	3	Apigenin; Luteolin; Scutellarein
12	KMT2A	50.55	3	Apigenin; Luteolin; Scutellarein
13	MAOA	47.96	4	Apigenin; Hyperin; Luteolin; Scutellarein
14	KDR	47.15	1	Luteolin
15	ABCG2	46.25	3	Apigenin; Luteolin; Scutellarein
16	AKT1	41.53	1	Luteolin
17	SLC2A1	40.84	2	Apigenin; Scutellarein
18	KIT	40.81	1	Scutellarein
19	MET	40.15	1	Luteolin
20	SYK	38.16	3	Apigenin; Luteolin; Scutellarein
21	CA4	35.35	4	Apigenin; Hyperin; Luteolin; Scutellarein
22	PKM	34.41	4	Apigenin; Hyperin; Luteolin; Scutellarein
23	PPARG	32.65	3	Apigenin; Luteolin; Scutellarein
24	CYP1B1	31.16	3	Apigenin; Luteolin; Scutellarein
25	FLT3	29.46	3	Apigenin; Luteolin; Scutellarein
26	AR	26.59	1	Apigenin
27	XDH	26.26	4	Apigenin; Hyperin; Luteolin; Scutellarein
28	SLCO2B1	25.49	3	Apigenin; Luteolin; Scutellarein
29	TYR	24.98	3	Apigenin;

				Luteolin; Scutellarein
30	ESR2	24.44	3	Apigenin; Luteolin; Scutellarein
31	SRC	24.32	1	Luteolin
32	PRKCA	23.67	4	Calceorioside B; Epimeredinoside A; Plantamajoside; Verbascoside
33	MMP9	23.65	5	Apigenin; Caffeic acid; Hyperin; Luteolin; Scutellarein
34	ALOX5	23.14	4	Apigenin; Caffeic acid; Luteolin; Scutellarein
35	CDK2	22.00	1	Luteolin
36	CDK6	21.75	3	Apigenin; Luteolin; Scutellarein
37	BCHE	20.43	1	Apigenin

Table S8. Repeat-resolved ligand–protein hydrogen-bond occupancy.

ligand	replicate	direction	acceptor	donor	occupancy	mean_distance_A	mean_angle_deg
VX7	pooled	ligdonor	SER_308@OG	LIG_1194@N16	87.27%	2.846	163.65
VX7	pooled	ligacc	LIG_1194@O13	LEU_932@N	85.23%	2.844	162.28
VX7	1	ligdonor	SER_308@OG	LIG_1194@N16	89.20%	2.838	162.54
VX7	1	ligacc	LIG_1194@O13	LEU_932@N	89.90%	2.836	161.66
VX7	2	ligdonor	SER_308@OG	LIG_1194@N16	87.50%	2.844	163.41
VX7	2	ligacc	LIG_1194@O13	LEU_932@N	83.20%	2.844	162.16
VX7	3	ligdonor	SER_308@OG	LIG_1194@N16	85.10%	2.857	165.06
VX7	3	ligacc	LIG_1194@O13	LEU_932@N	82.60%	2.851	163.06
Luteolin	pooled	ligdonor	GLU_874@OE1	LIG_1194@O2	98.27%	2.663	162.56
Luteolin	pooled	ligdonor	PHE_312@O	LIG_1194@O5	87.93%	2.734	161.72
Luteolin	pooled	ligdonor	LEU_927@O	LIG_1194@O4	53.40%	2.705	157.49
Luteolin	pooled	ligdonor	GLY_926@O	LIG_1194@O4	27.73%	2.710	160.52
Luteolin	1	ligdonor	GLU_874@OE1	LIG_1194@O2	98.70%	2.665	163.11

Luteolin	1	ligdonor	PHE_312@O	LIG_1194@O5	88.90%	2.723	161.08
Luteolin	1	ligdonor	LEU_927@O	LIG_1194@O4	83.50%	2.697	157.80
Luteolin	1	ligdonor	GLY_926@O	LIG_1194@O4	5.30%	2.719	161.71
Luteolin	2	ligdonor	GLU_874@OE1	LIG_1194@O2	97.70%	2.656	163.21
Luteolin	2	ligdonor	PHE_312@O	LIG_1194@O5	89.40%	2.733	161.02
Luteolin	2	ligdonor	LEU_927@O	LIG_1194@O4	54.60%	2.704	159.76
Luteolin	2	ligdonor	GLY_926@O	LIG_1194@O4	27.60%	2.718	159.83
Luteolin	2	ligacc	LIG_1194@O3	LEU_932@N	7.60%	2.900	150.44
Luteolin	3	ligdonor	GLU_874@OE1	LIG_1194@O2	98.40%	2.669	161.36
Luteolin	3	ligdonor	PHE_312@O	LIG_1194@O5	85.50%	2.745	163.10
Luteolin	3	ligdonor	GLY_926@O	LIG_1194@O4	50.30%	2.704	160.77
Luteolin	3	ligdonor	LEU_927@O	LIG_1194@O4	22.10%	2.738	150.70
Apigenin	pooled	ligdonor	GLU_874@OE1	LIG_1194@O4	14.47%	2.594	162.01
Apigenin	pooled	ligdonor	GLU_874@OE1	LIG_1194@O2	12.03%	2.612	167.35
Apigenin	pooled	ligdonor	LEU_927@O	LIG_1194@O4	11.43%	2.709	158.22
Apigenin	pooled	ligdonor	VAL_232@O	LIG_1194@O5	10.13%	2.785	162.02
Apigenin	pooled	ligdonor	GLY_926@O	LIG_1194@O4	8.60%	2.737	165.05
Apigenin	pooled	ligdonor	LEU_927@O	LIG_1194@O2	7.83%	2.716	165.63
Apigenin	pooled	ligdonor	PHE_229@O	LIG_1194@O5	7.30%	2.810	153.24
Apigenin	pooled	ligdonor	SER_308@OG	LIG_1194@O5	6.07%	2.825	163.17
Apigenin	1	ligdonor	LEU_315@O	LIG_1194@O4	8.70%	2.740	157.90
Apigenin	1	ligdonor	VAL_232@O	LIG_1194@O5	6.30%	2.772	163.40
Apigenin	1	ligdonor	PHE_229@O	LIG_1194@O5	5.50%	2.834	152.59
Apigenin	2	ligdonor	GLU_874@OE1	LIG_1194@O4	43.40%	2.594	162.01
Apigenin	2	ligdonor	VAL_232@O	LIG_1194@O5	23.00%	2.785	162.24
Apigenin	2	ligdonor	SER_308@OG	LIG_1194@O5	18.20%	2.825	163.17
Apigenin	2	ligdonor	GLU_874@OE2	LIG_1194@O4	9.50%	2.613	160.23
Apigenin	3	ligdonor	GLU_874@OE1	LIG_1194@O2	36.10%	2.612	167.35

Apigenin	3	ligdonor	LEU_927@O	LIG_1194@O4	33.80%	2.709	158.15
Apigenin	3	ligdonor	GLY_926@O	LIG_1194@O4	25.80%	2.737	165.05
Apigenin	3	ligdonor	LEU_927@O	LIG_1194@O2	22.80%	2.716	166.06
Apigenin	3	ligdonor	PHE_229@O	LIG_1194@O5	15.30%	2.795	153.72
Apigenin	3	ligacc	LIG_1194@O3	ARG_934@NE	6.60%	2.890	155.28
Apigenin	3	ligacc	LIG_1194@O2	ARG_934@NH1	5.50%	2.872	145.61
Scutellarein	pooled	ligdonor	LEU_927@O	LIG_1194@O3	33.00%	2.753	155.06
Scutellarein	pooled	ligdonor	GLU_874@OE1	LIG_1194@O4	21.63%	2.651	161.04
Scutellarein	pooled	ligdonor	GLU_874@OE1	LIG_1194@O3	12.80%	2.631	166.96
Scutellarein	pooled	ligdonor	GLU_874@OE2	LIG_1194@O4	9.67%	2.665	154.34
Scutellarein	pooled	ligdonor	GLY_926@O	LIG_1194@O2	5.10%	2.785	142.35
Scutellarein	1	ligdonor	LEU_927@O	LIG_1194@O3	40.00%	2.738	154.39
Scutellarein	1	ligdonor	GLU_874@OE1	LIG_1194@O4	39.50%	2.652	160.87
Scutellarein	1	ligdonor	GLU_874@OE2	LIG_1194@O4	28.20%	2.663	154.56
Scutellarein	1	ligdonor	GLU_874@OE1	LIG_1194@O3	22.80%	2.640	167.79
Scutellarein	1	ligdonor	GLY_926@O	LIG_1194@O2	8.30%	2.774	143.55
Scutellarein	1	ligdonor	GLU_874@OE2	LIG_1194@O3	7.50%	2.601	167.00
Scutellarein	2	ligdonor	LEU_927@O	LIG_1194@O3	59.00%	2.764	155.52
Scutellarein	2	ligdonor	GLU_874@OE1	LIG_1194@O4	25.40%	2.650	161.30
Scutellarein	2	ligdonor	GLU_874@OE1	LIG_1194@O3	15.60%	2.619	165.75
Scutellarein	2	ligdonor	GLY_926@O	LIG_1194@O2	7.00%	2.797	140.93

Table S9. Whole-trajectory ligand–CFTR heavy-atom contact occupancy.

ligand	replicate	Native CFTR residue	resname	any_heavy_atom_contact_occupancy
VX7	1	233	LEU	94.30%
VX7	1	236	PHE	87.40%
VX7	1	304	TYR	99.50%
VX7	1	305	PHE	100.00%
VX7	1	308	SER	100.00%
VX7	1	309	ALA	98.70%
VX7	1	312	PHE	100.00%

VX7	1	316	PHE	11.80%
VX7	1	931	GLY	100.00%
VX7	1	932	LEU	100.00%
VX7	1	933	PHE	99.30%
VX7	2	233	LEU	80.40%
VX7	2	236	PHE	84.50%
VX7	2	304	TYR	98.30%
VX7	2	305	PHE	100.00%
VX7	2	308	SER	100.00%
VX7	2	309	ALA	96.70%
VX7	2	312	PHE	100.00%
VX7	2	315	LEU	18.10%
VX7	2	316	PHE	25.20%
VX7	2	929	ALA	14.20%
VX7	2	931	GLY	100.00%
VX7	2	932	LEU	100.00%
VX7	2	933	PHE	97.80%
VX7	3	233	LEU	91.00%
VX7	3	236	PHE	78.00%
VX7	3	304	TYR	99.10%
VX7	3	305	PHE	100.00%
VX7	3	308	SER	100.00%
VX7	3	309	ALA	98.90%
VX7	3	312	PHE	100.00%
VX7	3	315	LEU	15.80%
VX7	3	316	PHE	35.50%
VX7	3	931	GLY	100.00%
VX7	3	932	LEU	100.00%
VX7	3	933	PHE	98.80%
VX7	replicate_mean	233	LEU	88.57%
VX7	replicate_mean	236	PHE	83.30%
VX7	replicate_mean	304	TYR	98.97%
VX7	replicate_mean	305	PHE	100.00%
VX7	replicate_mean	308	SER	100.00%
VX7	replicate_mean	309	ALA	98.10%
VX7	replicate_mean	312	PHE	100.00%
VX7	replicate_mean	315	LEU	16.95%
VX7	replicate_mean	316	PHE	24.17%
VX7	replicate_mean	931	GLY	100.00%
VX7	replicate_mean	932	LEU	100.00%
VX7	replicate_mean	933	PHE	98.63%
Luteolin	1	312	PHE	100.00%
Luteolin	1	313	SER	69.90%

Luteolin	1	315	LEU	47.90%
Luteolin	1	316	PHE	82.60%
Luteolin	1	874	GLU	100.00%
Luteolin	1	878	CYS	13.50%
Luteolin	1	926	GLY	47.90%
Luteolin	1	927	LEU	100.00%
Luteolin	1	928	LEU	68.70%
Luteolin	1	929	ALA	99.80%
Luteolin	1	931	GLY	100.00%
Luteolin	1	932	LEU	96.40%
Luteolin	1	933	PHE	98.00%
Luteolin	1	934	ARG	48.20%
Luteolin	2	312	PHE	100.00%
Luteolin	2	313	SER	60.60%
Luteolin	2	316	PHE	98.10%
Luteolin	2	874	GLU	99.90%
Luteolin	2	926	GLY	50.40%
Luteolin	2	927	LEU	100.00%
Luteolin	2	928	LEU	92.50%
Luteolin	2	929	ALA	99.90%
Luteolin	2	931	GLY	100.00%
Luteolin	2	932	LEU	99.90%
Luteolin	2	933	PHE	98.40%
Luteolin	2	934	ARG	48.20%
Luteolin	3	229	PHE	36.30%
Luteolin	3	312	PHE	100.00%
Luteolin	3	313	SER	69.60%
Luteolin	3	315	LEU	31.10%
Luteolin	3	316	PHE	95.80%
Luteolin	3	874	GLU	100.00%
Luteolin	3	926	GLY	93.00%
Luteolin	3	927	LEU	100.00%
Luteolin	3	928	LEU	81.10%
Luteolin	3	929	ALA	99.90%
Luteolin	3	931	GLY	100.00%
Luteolin	3	932	LEU	99.10%
Luteolin	3	933	PHE	99.10%
Luteolin	3	934	ARG	51.00%
Luteolin	replicate_mean	229	PHE	14.63%
Luteolin	replicate_mean	312	PHE	100.00%
Luteolin	replicate_mean	313	SER	66.70%
Luteolin	replicate_mean	315	LEU	29.53%
Luteolin	replicate_mean	316	PHE	92.17%
Luteolin	replicate_mean	874	GLU	99.97%
Luteolin	replicate_mean	926	GLY	63.77%
Luteolin	replicate_mean	927	LEU	100.00%
Luteolin	replicate_mea	928	LEU	80.77%

	n			
Luteolin	replicate_mean	929	ALA	99.87%
Luteolin	replicate_mean	931	GLY	100.00%
Luteolin	replicate_mean	932	LEU	98.47%
Luteolin	replicate_mean	933	PHE	98.50%
Luteolin	replicate_mean	934	ARG	49.13%
Apigenin	1	229	PHE	33.30%
Apigenin	1	232	VAL	42.30%
Apigenin	1	233	LEU	42.10%
Apigenin	1	236	PHE	43.10%
Apigenin	1	305	PHE	34.00%
Apigenin	1	312	PHE	15.70%
Apigenin	1	315	LEU	46.50%
Apigenin	1	316	PHE	53.60%
Apigenin	1	319	PHE	50.70%
Apigenin	1	932	LEU	27.30%
Apigenin	2	232	VAL	38.80%
Apigenin	2	233	LEU	40.00%
Apigenin	2	236	PHE	43.40%
Apigenin	2	304	TYR	37.20%
Apigenin	2	305	PHE	89.70%
Apigenin	2	308	SER	33.30%
Apigenin	2	312	PHE	64.90%
Apigenin	2	315	LEU	49.00%
Apigenin	2	316	PHE	17.30%
Apigenin	2	870	VAL	13.30%
Apigenin	2	874	GLU	54.30%
Apigenin	2	926	GLY	53.40%
Apigenin	2	927	LEU	53.70%
Apigenin	2	929	ALA	54.20%
Apigenin	2	930	LEU	27.40%
Apigenin	2	931	GLY	60.10%
Apigenin	2	932	LEU	93.10%
Apigenin	2	933	PHE	59.90%
Apigenin	2	934	ARG	53.30%
Apigenin	3	229	PHE	34.00%
Apigenin	3	232	VAL	36.40%
Apigenin	3	233	LEU	36.30%
Apigenin	3	236	PHE	35.10%
Apigenin	3	305	PHE	35.10%
Apigenin	3	312	PHE	87.60%
Apigenin	3	315	LEU	24.00%
Apigenin	3	316	PHE	15.60%
Apigenin	3	870	VAL	29.20%
Apigenin	3	874	GLU	56.60%
Apigenin	3	926	GLY	63.60%
Apigenin	3	927	LEU	60.80%
Apigenin	3	928	LEU	38.40%
Apigenin	3	929	ALA	63.30%
Apigenin	3	931	GLY	63.60%

Apigenin	3	932	LEU	86.50%
Apigenin	3	933	PHE	60.30%
Apigenin	3	934	ARG	59.90%
Apigenin	replicate_mean	229	PHE	25.13%
Apigenin	replicate_mean	232	VAL	39.17%
Apigenin	replicate_mean	233	LEU	39.47%
Apigenin	replicate_mean	236	PHE	40.53%
Apigenin	replicate_mean	304	TYR	12.70%
Apigenin	replicate_mean	305	PHE	52.93%
Apigenin	replicate_mean	308	SER	17.45%
Apigenin	replicate_mean	312	PHE	56.07%
Apigenin	replicate_mean	315	LEU	39.83%
Apigenin	replicate_mean	316	PHE	28.83%
Apigenin	replicate_mean	319	PHE	50.70%
Apigenin	replicate_mean	870	VAL	21.25%
Apigenin	replicate_mean	874	GLU	55.45%
Apigenin	replicate_mean	926	GLY	58.50%
Apigenin	replicate_mean	927	LEU	57.25%
Apigenin	replicate_mean	928	LEU	20.90%
Apigenin	replicate_mean	929	ALA	39.20%
Apigenin	replicate_mean	930	LEU	18.00%
Apigenin	replicate_mean	931	GLY	42.63%
Apigenin	replicate_mean	932	LEU	68.97%
Apigenin	replicate_mean	933	PHE	41.00%
Apigenin	replicate_mean	934	ARG	56.60%
Scutellarein	1	305	PHE	18.10%
Scutellarein	1	311	PHE	53.30%
Scutellarein	1	312	PHE	99.50%
Scutellarein	1	316	PHE	83.00%
Scutellarei	1	874	GLU	80.50%

n				
Scutellarein	1	926	GLY	94.60%
Scutellarein	1	927	LEU	86.90%
Scutellarein	1	928	LEU	43.40%
Scutellarein	1	929	ALA	80.20%
Scutellarein	1	931	GLY	98.70%
Scutellarein	1	932	LEU	87.10%
Scutellarein	1	933	PHE	62.00%
Scutellarein	1	934	ARG	74.60%
Scutellarein	2	305	PHE	13.20%
Scutellarein	2	311	PHE	54.90%
Scutellarein	2	312	PHE	99.90%
Scutellarein	2	315	LEU	10.90%
Scutellarein	2	316	PHE	78.50%
Scutellarein	2	874	GLU	66.70%
Scutellarein	2	926	GLY	96.50%
Scutellarein	2	927	LEU	93.40%
Scutellarein	2	928	LEU	66.50%
Scutellarein	2	929	ALA	96.80%
Scutellarein	2	930	LEU	37.20%
Scutellarein	2	931	GLY	100.00%
Scutellarein	2	932	LEU	60.30%
Scutellarein	2	933	PHE	85.00%
Scutellarein	2	934	ARG	24.00%
Scutellarein	3	305	PHE	22.60%
Scutellarein	3	312	PHE	47.90%
Scutellarein	3	870	VAL	52.50%
Scutellarein	3	874	GLU	65.00%
Scutellarei	3	929	ALA	96.30%

n				
Scutellarein	3	931	GLY	96.70%
Scutellarein	3	932	LEU	83.90%
Scutellarein	3	933	PHE	96.40%
Scutellarein	3	934	ARG	82.40%
Scutellarein	replicate_mean	305	PHE	17.97%
Scutellarein	replicate_mean	311	PHE	54.10%
Scutellarein	replicate_mean	312	PHE	82.43%
Scutellarein	replicate_mean	316	PHE	55.53%
Scutellarein	replicate_mean	870	VAL	26.30%
Scutellarein	replicate_mean	874	GLU	70.73%
Scutellarein	replicate_mean	926	GLY	63.77%
Scutellarein	replicate_mean	927	LEU	60.97%
Scutellarein	replicate_mean	928	LEU	54.95%
Scutellarein	replicate_mean	929	ALA	91.10%
Scutellarein	replicate_mean	930	LEU	14.73%
Scutellarein	replicate_mean	931	GLY	98.47%
Scutellarein	replicate_mean	932	LEU	77.10%
Scutellarein	replicate_mean	933	PHE	81.13%
Scutellarein	replicate_mean	934	ARG	60.33%

Table S10. Protein and ligand RMSD summaries for individual trajectories and across-trajectory means.

Ligand	Trajectory	Protein RMSD (Å)	Ligand RMSD (Å)	Frames
Ivacaftor	1	2.088	1.789	1000
Ivacaftor	2	1.494	2.249	1000
Ivacaftor	3	1.618	2.176	1000
Ivacaftor	Mean ± SD	1.73 ± 0.31	2.07 ± 0.25	3 × 1000
Luteolin	1	1.886	2.064	1000
Luteolin	2	1.932	1.574	1000
Luteolin	3	2.237	1.741	1000
Luteolin	Mean ± SD	2.02 ± 0.19	1.79 ± 0.25	3 × 1000

Apigenin	1	1.754	9.348	1000
Apigenin	2	1.701	8.388	1000
Apigenin	3	1.813	9.004	1000
Apigenin	Mean ± SD	1.76 ± 0.06	8.91 ± 0.49	3 × 1000
Scutellarein	1	1.800	7.083	1000
Scutellarein	2	2.017	7.663	1000
Scutellarein	3	1.722	7.331	1000
Scutellarein	Mean ± SD	1.85 ± 0.15	7.36 ± 0.29	3 × 1000

Table S11. Membrane molecular-dynamics system composition and protocol.

Parameter	Value
Protein template	PDB 6O2P (ivacaftor-bound human CFTR cryo-EM structure); porcine CFTR homology model built on this template
Protein force field	ff19SB
Membrane lipid	POPC (Lipid21 force field); 248 molecules total (124 per leaflet)
Ligand parameters	GAFF2 with AM1-BCC charges (apigenin/luteolin/scutellarein); ivacaftor pose taken directly from 6O2P and not re-docked
ATP parameters	Literature AMBER ATP parameters (2 ATP + 2 Mg2+ per system at NBD sites)
Water model	OPC; 71146 waters per system
Ions	~0.15 M KCl; 193 K+ / 217 Cl- per system (24 extra Cl- neutralize +24 solute charge)
Simulation engine	AMBER 25 (pmemd)
Ensemble	Semi-isotropic NPT (ntp=3), constant surface tension (csurften=3, ninterface=2)
Equilibration	3 minimizations; 1 ns NVT heating 0-300 K; 6-stage membrane equilibration totalling 21 ns (22 ns including heating)
Production	3 separately executed trajectories per ligand; all production runs started from the same equilibrated eq6.rst7 restart with irest=1 and ntx=5 and therefore inherited the same initial velocities; 200 ns planned per trajectory
Primary comparative analysis window	0-120 ns from each of 12 trajectories; 1000 uniformly sampled frames per trajectory (120 ps spacing); 360 ns per ligand and 1.44 microseconds total
Post-processing burn-in	None; the complete 0-120 ns production window was analysed as specified
Analysis replication	Metrics calculated separately for 3 trajectories per ligand and summarized across trajectories; trajectory frames were not treated as independent replicates
Trajectory processing	cpptraj from AmberTools 25; protein backbone defined as CA/C/N/O and per-residue RMSF by C-alpha
Ligand-protein contacts	MDAnalysis 2.4.3 dynamic any-heavy-atom contacts, 4.5 A cutoff, by protein residue; includes contacts formed after pose migration
Ligand-protein H-bonds	cpptraj hbond, default distance (3.0 A heavy-atom) and angle (135 deg) cutoffs, donor/acceptor roles split explicitly for ligand and protein

Table S12. Consecutive 40 ns protein and ligand RMSD block summaries.

ligand	replicate	metric	block_40ns	mean_A	SD_A	frames
VX7	1	rmsd_protein	1	1.671	0.299	334
VX7	1	rmsd_protein	2	2.244	0.139	333
VX7	1	rmsd_protein	3	2.350	0.181	333
VX7	1	rmsd_ligand	1	1.671	0.465	334
VX7	1	rmsd_ligand	2	1.618	0.351	333

VX7	1	rmsd_ligand	3	2.076	0.454	333
VX7	2	rmsd_protein	1	1.351	0.147	334
VX7	2	rmsd_protein	2	1.443	0.103	333
VX7	2	rmsd_protein	3	1.687	0.093	333
VX7	2	rmsd_ligand	1	1.670	0.418	334
VX7	2	rmsd_ligand	2	2.523	0.271	333
VX7	2	rmsd_ligand	3	2.556	0.353	333
VX7	3	rmsd_protein	1	1.344	0.212	334
VX7	3	rmsd_protein	2	1.697	0.122	333
VX7	3	rmsd_protein	3	1.815	0.091	333
VX7	3	rmsd_ligand	1	1.780	0.428	334
VX7	3	rmsd_ligand	2	2.390	0.389	333
VX7	3	rmsd_ligand	3	2.359	0.433	333
Luteolin	1	rmsd_protein	1	1.685	0.323	334
Luteolin	1	rmsd_protein	2	1.991	0.113	333
Luteolin	1	rmsd_protein	3	1.983	0.108	333
Luteolin	1	rmsd_ligand	1	1.737	0.587	334
Luteolin	1	rmsd_ligand	2	2.463	0.580	333
Luteolin	1	rmsd_ligand	3	1.992	0.466	333
Luteolin	2	rmsd_protein	1	1.674	0.299	334
Luteolin	2	rmsd_protein	2	2.009	0.128	333
Luteolin	2	rmsd_protein	3	2.113	0.117	333
Luteolin	2	rmsd_ligand	1	1.190	0.434	334
Luteolin	2	rmsd_ligand	2	1.517	0.454	333
Luteolin	2	rmsd_ligand	3	2.018	0.373	333
Luteolin	3	rmsd_protein	1	1.867	0.367	334
Luteolin	3	rmsd_protein	2	2.349	0.145	333
Luteolin	3	rmsd_protein	3	2.495	0.137	333
Luteolin	3	rmsd_ligand	1	1.663	0.459	334
Luteolin	3	rmsd_ligand	2	1.738	0.343	333
Luteolin	3	rmsd_ligand	3	1.823	0.335	333
Apigenin	1	rmsd_protein	1	1.490	0.209	334
Apigenin	1	rmsd_protein	2	1.837	0.147	333
Apigenin	1	rmsd_protein	3	1.937	0.125	333
Apigenin	1	rmsd_ligand	1	1.789	0.586	334
Apigenin	1	rmsd_ligand	2	10.639	5.420	333
Apigenin	1	rmsd_ligand	3	15.640	0.750	333
Apigenin	2	rmsd_protein	1	1.572	0.260	334
Apigenin	2	rmsd_protein	2	1.713	0.121	333
Apigenin	2	rmsd_protein	3	1.817	0.117	333
Apigenin	2	rmsd_ligand	1	2.468	0.638	334
Apigenin	2	rmsd_ligand	2	9.747	4.048	333
Apigenin	2	rmsd_ligand	3	12.968	0.716	333
Apigenin	3	rmsd_protein	1	1.600	0.209	334
Apigenin	3	rmsd_protein	2	1.835	0.125	333
Apigenin	3	rmsd_protein	3	2.005	0.124	333
Apigenin	3	rmsd_ligand	1	2.252	0.536	334
Apigenin	3	rmsd_ligand	2	11.790	2.441	333
Apigenin	3	rmsd_ligand	3	12.989	0.500	333
Scutellarein	1	rmsd_protein	1	1.525	0.232	334
Scutellarein	1	rmsd_protein	2	1.834	0.133	333
Scutellarein	1	rmsd_protein	3	2.043	0.152	333
Scutellarein	1	rmsd_ligand	1	6.734	1.263	334
Scutellarein	1	rmsd_ligand	2	7.154	0.452	333
Scutellarein	1	rmsd_ligand	3	7.362	0.349	333

Scutellarein	2	rmsd_protein	1	1.576	0.346	334
Scutellarein	2	rmsd_protein	2	2.008	0.163	333
Scutellarein	2	rmsd_protein	3	2.469	0.115	333
Scutellarein	2	rmsd_ligand	1	7.301	1.479	334
Scutellarein	2	rmsd_ligand	2	7.884	0.323	333
Scutellarein	2	rmsd_ligand	3	7.805	0.311	333
Scutellarein	3	rmsd_protein	1	1.380	0.256	334
Scutellarein	3	rmsd_protein	2	1.914	0.149	333
Scutellarein	3	rmsd_protein	3	1.874	0.124	333
Scutellarein	3	rmsd_ligand	1	6.753	1.197	334
Scutellarein	3	rmsd_ligand	2	6.655	0.381	333
Scutellarein	3	rmsd_ligand	3	8.586	1.933	333

Table S13A. Sampled frame fractions of apigenin State A and State B in each trajectory.

replicate	state	frames	sampled frame fraction
1	State A	434	43.4%
1	State B	566	56.6%
2	State A	438	43.8%
2	State B	562	56.2%
3	State A	364	36.4%
3	State B	636	63.6%

Table S13B. Sustained apigenin State B transition and post-transition persistence.

**Trajectory**	**Transition time (ns)**	**Persistence to final frame (ns)**	**Subsequent return to State A**
1	52.08	67.80	No
2	54.00	65.88	No
3	43.68	76.20	No

Table S14. Simulation-topology to native porcine CFTR mapping for residues represented in the state-resolved contact analysis.

Topology residue	Pig CFTR chain	Native pig CFTR residue	Residue name
228	A	228	ALA
229	A	229	PHE
232	A	232	VAL
233	A	233	LEU
235	A	235	LEU
236	A	236	PHE
237	A	237	GLN
240	A	240	LEU
304	A	304	TYR
305	A	305	PHE
308	A	308	SER
309	A	309	ALA
311	A	311	PHE
312	A	312	PHE
313	A	313	SER
314	A	314	GLY
315	A	315	LEU
316	A	316	PHE
319	A	319	PHE
323	A	323	LEU

635	A	870	VAL
636	A	871	PHE
639	A	874	GLU
643	A	878	CYS
690	A	925	ASP
691	A	926	GLY
692	A	927	LEU
693	A	928	LEU
694	A	929	ALA
695	A	930	LEU
696	A	931	GLY
697	A	932	LEU
698	A	933	PHE
699	A	934	ARG

Table S15. State-resolved ligand–CFTR heavy-atom contact occupancy.

Ensemble	Topology residue	Native pig residue	Residue	Contact frames	State frames	Occupancy (%)
Ivacaftor	229	229	PHE	4	3000	0.13
Ivacaftor	233	233	LEU	2657	3000	88.57
Ivacaftor	236	236	PHE	2499	3000	83.30
Ivacaftor	237	237	GLN	1	3000	0.03
Ivacaftor	304	304	TYR	2969	3000	98.97
Ivacaftor	305	305	PHE	3000	3000	100.00
Ivacaftor	308	308	SER	3000	3000	100.00
Ivacaftor	309	309	ALA	2943	3000	98.10
Ivacaftor	311	311	PHE	6	3000	0.20
Ivacaftor	312	312	PHE	3000	3000	100.00
Ivacaftor	313	313	SER	3	3000	0.10
Ivacaftor	315	315	LEU	339	3000	11.30
Ivacaftor	316	316	PHE	725	3000	24.17
Ivacaftor	639	874	GLU	1	3000	0.03
Ivacaftor	691	926	GLY	57	3000	1.90
Ivacaftor	692	927	LEU	2	3000	0.07
Ivacaftor	694	929	ALA	143	3000	4.77
Ivacaftor	695	930	LEU	38	3000	1.27
Ivacaftor	696	931	GLY	3000	3000	100.00
Ivacaftor	697	932	LEU	3000	3000	100.00
Ivacaftor	698	933	PHE	2959	3000	98.63
Luteolin	229	229	PHE	439	3000	14.63
Luteolin	233	233	LEU	28	3000	0.93
Luteolin	305	305	PHE	14	3000	0.47
Luteolin	312	312	PHE	3000	3000	100.00
Luteolin	313	313	SER	2001	3000	66.70
Luteolin	315	315	LEU	886	3000	29.53
Luteolin	316	316	PHE	2765	3000	92.17
Luteolin	636	871	PHE	1	3000	0.03
Luteolin	639	874	GLU	2999	3000	99.97
Luteolin	643	878	CYS	206	3000	6.87
Luteolin	690	925	ASP	23	3000	0.77
Luteolin	691	926	GLY	1913	3000	63.77
Luteolin	692	927	LEU	3000	3000	100.00
Luteolin	693	928	LEU	2423	3000	80.77
Luteolin	694	929	ALA	2996	3000	99.87

Luteolin	695	930	LEU	197	3000	6.57
Luteolin	696	931	GLY	3000	3000	100.00
Luteolin	697	932	LEU	2954	3000	98.47
Luteolin	698	933	PHE	2955	3000	98.50
Luteolin	699	934	ARG	1474	3000	49.13
Apigenin State A	228	228	ALA	22	1236	1.78
Apigenin State A	229	229	PHE	750	1236	60.68
Apigenin State A	232	232	VAL	1175	1236	95.06
Apigenin State A	233	233	LEU	1180	1236	95.47
Apigenin State A	235	235	LEU	25	1236	2.02
Apigenin State A	236	236	PHE	1210	1236	97.90
Apigenin State A	240	240	LEU	2	1236	0.16
Apigenin State A	304	304	TYR	4	1236	0.32
Apigenin State A	305	305	PHE	1091	1236	88.27
Apigenin State A	308	308	SER	16	1236	1.29
Apigenin State A	309	309	ALA	12	1236	0.97
Apigenin State A	312	312	PHE	509	1236	41.18
Apigenin State A	313	313	SER	2	1236	0.16
Apigenin State A	316	316	PHE	77	1236	6.23
Apigenin State A	696	931	GLY	46	1236	3.72
Apigenin State A	697	932	LEU	857	1236	69.34
Apigenin State A	698	933	PHE	46	1236	3.72
Apigenin State B	229	229	PHE	4	1764	0.23
Apigenin State B	233	233	LEU	4	1764	0.23
Apigenin State B	236	236	PHE	6	1764	0.34
Apigenin State B	304	304	TYR	377	1764	21.37
Apigenin State B	305	305	PHE	497	1764	28.17
Apigenin State B	308	308	SER	333	1764	18.88
Apigenin State B	309	309	ALA	33	1764	1.87
Apigenin State B	311	311	PHE	15	1764	0.85
Apigenin	312	312	PHE	1173	1764	66.50

State B						
Apigenin State B	314	314	GLY	1	1764	0.06
Apigenin State B	315	315	LEU	1195	1764	67.74
Apigenin State B	316	316	PHE	788	1764	44.67
Apigenin State B	319	319	PHE	507	1764	28.74
Apigenin State B	323	323	LEU	38	1764	2.15
Apigenin State B	635	870	VAL	425	1764	24.09
Apigenin State B	639	874	GLU	1109	1764	62.87
Apigenin State B	690	925	ASP	1	1764	0.06
Apigenin State B	691	926	GLY	1170	1764	66.33
Apigenin State B	692	927	LEU	1145	1764	64.91
Apigenin State B	693	928	LEU	418	1764	23.70
Apigenin State B	694	929	ALA	1176	1764	66.67
Apigenin State B	695	930	LEU	360	1764	20.41
Apigenin State B	696	931	GLY	1233	1764	69.90
Apigenin State B	697	932	LEU	1212	1764	68.71
Apigenin State B	698	933	PHE	1184	1764	67.12
Apigenin State B	699	934	ARG	1132	1764	64.17
Scutellarein	229	229	PHE	108	3000	3.60
Scutellarein	233	233	LEU	59	3000	1.97
Scutellarein	236	236	PHE	2	3000	0.07
Scutellarein	304	304	TYR	38	3000	1.27
Scutellarein	305	305	PHE	539	3000	17.97
Scutellarein	308	308	SER	49	3000	1.63
Scutellarein	309	309	ALA	1	3000	0.03
Scutellarein	311	311	PHE	1082	3000	36.07
Scutellarein	312	312	PHE	2473	3000	82.43
Scutellarein	313	313	SER	3	3000	0.10
Scutellarein	315	315	LEU	172	3000	5.73
Scutellarein	316	316	PHE	1666	3000	55.53
Scutellarein	635	870	VAL	526	3000	17.53
Scutellarein	636	871	PHE	2	3000	0.07
Scutellarein	639	874	GLU	2122	3000	70.73
Scutellarein	690	925	ASP	81	3000	2.70
Scutellarein	691	926	GLY	1913	3000	63.77
Scutellarein	692	927	LEU	1829	3000	60.97
Scutellarein	693	928	LEU	1099	3000	36.63
Scutellarein	694	929	ALA	2733	3000	91.10

Scutellarein	695	930	LEU	442	3000	14.73
Scutellarein	696	931	GLY	2954	3000	98.47
Scutellarein	697	932	LEU	2313	3000	77.10
Scutellarein	698	933	PHE	2434	3000	81.13
Scutellarein	699	934	ARG	1810	3000	60.33

Table S16. State-resolved TM8 hinge-angle and TM5–TM8 pocket-distance summaries.

Ensemble	Trajectory	Frames	TM8 hinge angle (°)	TM5–TM8 pocket distance (Å)
Ivacaftor	1	1000	42.94 ± 2.48	12.83 ± 0.47
Ivacaftor	2	1000	41.52 ± 2.83	13.18 ± 0.39
Ivacaftor	3	1000	43.47 ± 2.47	13.44 ± 0.40
Luteolin	1	1000	38.39 ± 2.65	12.95 ± 0.42
Luteolin	2	1000	38.09 ± 2.62	12.91 ± 0.33
Luteolin	3	1000	40.82 ± 2.67	12.39 ± 0.42
Apigenin State A	1	434	41.87 ± 2.24	13.69 ± 0.38
Apigenin State B	1	566	41.31 ± 2.39	13.79 ± 0.48
Apigenin State A	2	438	41.87 ± 2.82	12.26 ± 0.71
Apigenin State B	2	562	44.05 ± 2.87	13.65 ± 0.64
Apigenin State A	3	364	40.60 ± 2.27	13.15 ± 0.44
Apigenin State B	3	636	40.92 ± 2.50	13.10 ± 0.56
Scutellarein	1	1000	41.88 ± 3.05	13.55 ± 0.39
Scutellarein	2	1000	42.49 ± 2.56	13.35 ± 0.42
Scutellarein	3	1000	41.90 ± 2.88	13.79 ± 0.40
Ivacaftor	Across trajectories	3 trajectories	42.64 ± 1.01	13.15 ± 0.31
Luteolin	Across trajectories	3 trajectories	39.10 ± 1.50	12.75 ± 0.31
Apigenin State A	Across trajectories	3 trajectories	41.45 ± 0.73	13.04 ± 0.72
Apigenin State B	Across trajectories	3 trajectories	42.09 ± 1.71	13.51 ± 0.37
Scutellarein	Across trajectories	3 trajectories	42.09 ± 0.35	13.56 ± 0.22

Table S17. Atom counts and periodic-cell dimensions of the equilibrated membrane simulation systems.

System	Total atoms	Equilibrated cell dimensions, X × Y × Z (Å)
Ivacaftor	337,707	105.21 × 105.21 × 237.49
Luteolin	337,681	105.08 × 105.08 × 238.13
Apigenin	337,680	105.79 × 105.79 × 234.63
Scutellarein	337,681	105.73 × 105.73 × 235.11

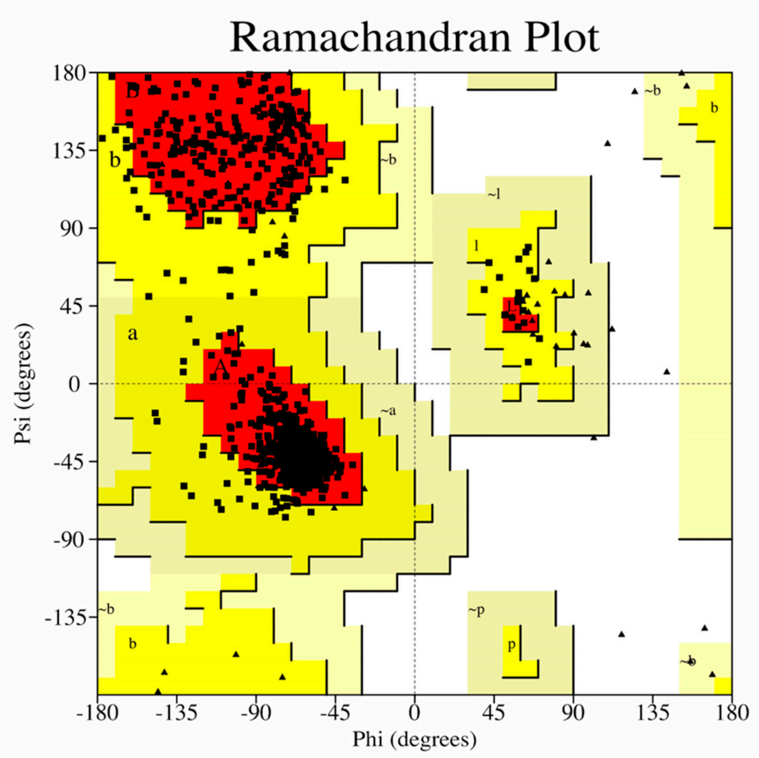

Figure S1. PROCHECK Ramachandran plot of the porcine CFTR model. A total of 92.6% of the evaluated residues were located in the most favoured regions and 7.4% in additionally allowed regions, with no residues in disallowed regions.

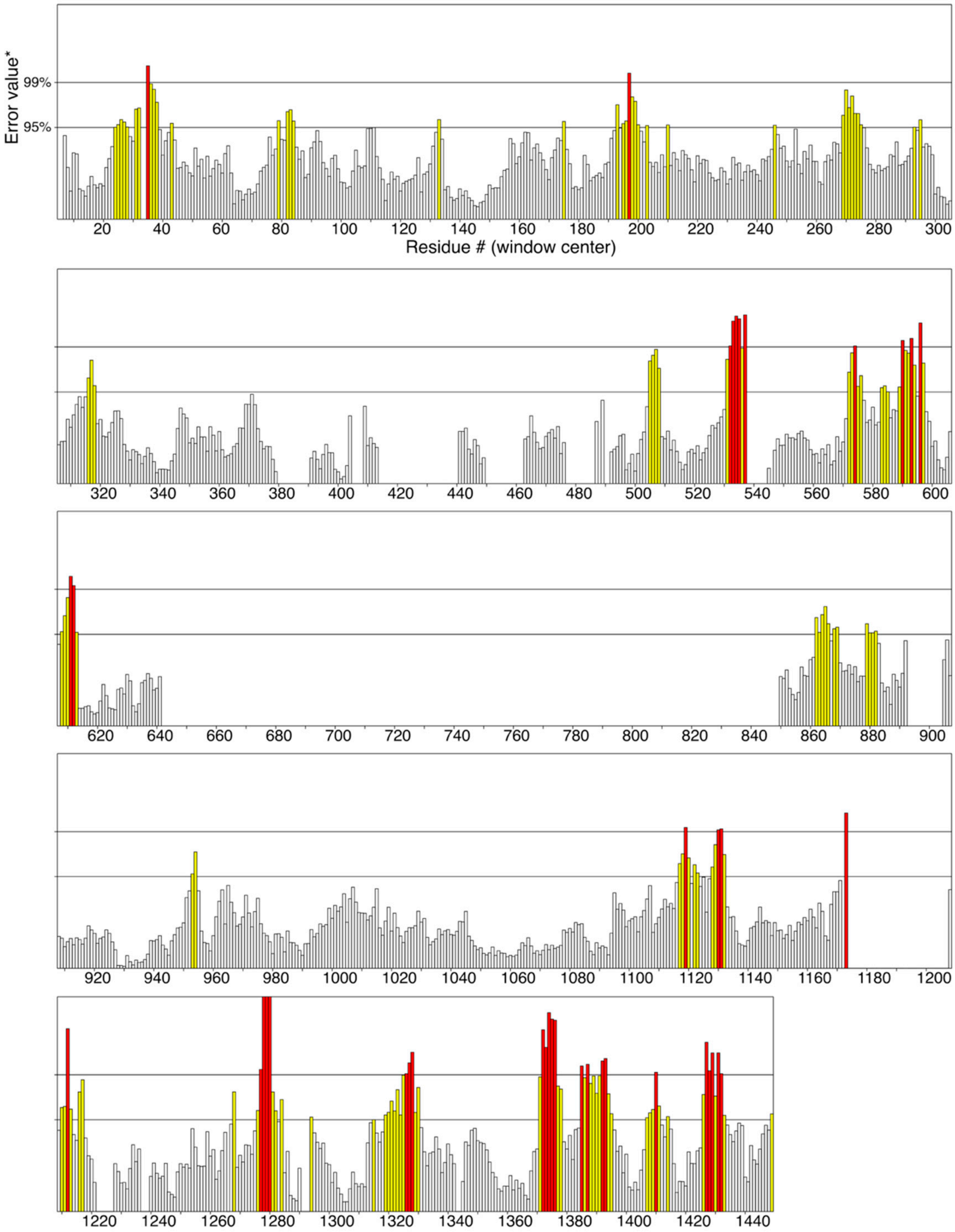

Figure S2. ERRAT analysis of the porcine CFTR model. The analysis of non-bonded atomic interactions yielded an Overall Quality Factor of 85.30.

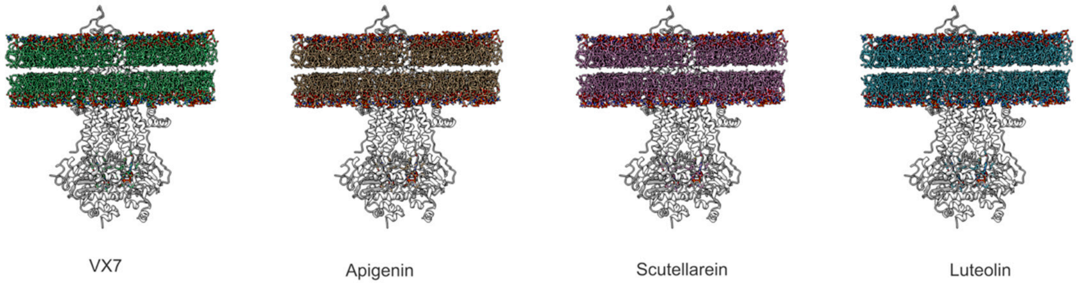

Figure S3. Equilibrated porcine CFTR membrane simulation systems. CFTR is shown as a cartoon, POPC molecules as sticks, the bound ligands and ATP molecules as sticks, and Mg^{2+} ions as spheres. Water molecules and free K^+ and Cl^- ions are omitted for visual clarity but were explicitly included in all simulations. Each system contained 248 POPC molecules, with 124 molecules in each leaflet.

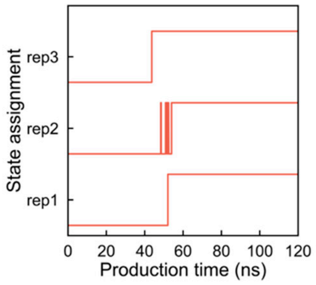

Figure S4. Apigenin state assignments across the three separately propagated trajectories. Each trajectory showed a sustained transition from State A to State B. After the indicated transition, State B persisted to the final analysed frame without returning to State A.
